# Intracellular TAS2Rs act as a gatekeeper for the excretion of harmful substances via ABCB1 in keratinocytes

**DOI:** 10.1096/fba.2024-00074

**Published:** 2024-08-27

**Authors:** Sazanami Mori, Natsuki Nakamura, Ayane Fuchigami, Satoshi Yoshimoto, Moe Sakakibara, Toshiyuki Ozawa, Junken Aoki, Asuka Inoue, Hayakazu Sumida, Hideya Ando, Motonao Nakamura

**Affiliations:** ^1^ Department of Bioscience, Graduate School of Life Science Okayama University of Science Okayama Japan; ^2^ Department of Dermatology, Faculty of Medicine The University of Tokyo Bunkyo‐ku Tokyo Japan; ^3^ Pharmaco‐Physiology and Kinetics Collaborate Research Division, Graduate School of Medicine Osaka Metropolitan University Osaka Japan; ^4^ Department of Health Chemistry, Graduate School of Pharmaceutical Science The University of Tokyo Bunkyo‐ku Tokyo Japan; ^5^ Japan Agency for Medical Research and Development Core Research for Evolutional Science and Technology Chiyoda‐ku Tokyo Japan; ^6^ Department of Molecular and Cellular Biochemistry, Graduate School of Pharmaceutical Sciences Tohoku University Sendai Miyagi Japan

**Keywords:** ABCB1, bitter taste receptors, host defense, intracellular location, keratinocytes: GPCR

## Abstract

Bitter taste receptors (TAS2Rs) are not only expressed in the oral cavity but also in skin. Extraoral TAS2Rs are thought to be involved in non‐taste perception and tissue‐specific functions. Keratinocytes that express TAS2Rs in the skin provide a first‐line defense against external threats. However, the functional roles of these receptors in host defense remain unclear. Here, we demonstrated the sensory role of intracellularly located TAS2Rs against toxic substances in keratinocytes. Although many G protein‐coupled receptors elicit signals from the surface, TAS2Rs were found to localize intracellularly, possibly to the ER, in human keratinocytes and HaCaT cells. TAS2R38, one of the TAS2R members, activated the G_α12/13_/RhoA/ROCK/p38 MAP kinase/NF‐κB pathway upon stimulation by phenylthiocarbamide (PTC), an agonist for this receptor, leading to the production of ABC transporters, such as ABCB1, in these cells. Notably, treatment with bitter compounds, such as PTC and saccharin, induced the upregulation of ABCB1 in HaCaT cells. Mechanistically, intracellular TAS2R38 and its downstream signaling G_α12/13_/RhoA/ROCK/p38 MAP kinase/NF‐κB pathway were identified to be responsible for the above effect. Pretreatment with PTC prevented the accumulation of rhodamine 123 because of its excretion via ABCB1. Furthermore, pretreatment with PTC or saccharin counteracted the effect of the toxic compound, diphenhydramine, and pretreated HaCaT cells were found to proliferate faster than untreated cells. This anti‐toxic effect was suppressed by treatment with verapamil, an ABCB1 inhibitor, indicating that enhanced ABCB1 helps clear toxic substances. Altogether, harmless activators of TAS2Rs may be promising drugs that enhance the excretion of toxic substances from the human skin.

## Graphical abstract

Schematic model of the excretion of harmful substances by the activation of the intracellular TAS2Rs. Human keratinocytes that express bitter taste receptors (TAS2Rs) in the skin provide a first‐line defense against external threats. Although many G protein‐coupled receptors elicit signals from the surface, TAS2Rs were found to localize intracellularly, possibly to the ER, in human keratinocytes and HaCaT cells. Activation of intracellular TAS2Rs enhances the expressions of ABC transporters, such as ABCB1, leading to the prevention of cellular damage. For example, pretreatment with bitter substances, such as phenylthiocarbamide or saccharin, counteracted the effect of the toxic compound, diphenhydramine, and pretreated HaCaT cells were found to proliferate faster than untreated cells. Mechanistically, intracellular TAS2Rs and its downstream signaling G_α12/13_/RhoA/ROCK/p38 MAP kinase/NF‐κB pathway were identified to be responsible for the above effect. Altogether, harmless activators of TAS2Rs may be promising drugs that enhance the excretion of toxic substances from the human skin.

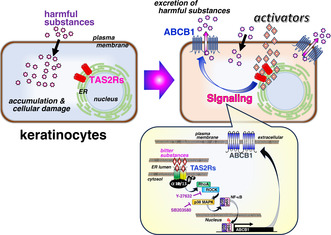

**Abbreviations**
ABCB1: ATP binding cassette transporter type‐B1
AD: atopic dermatitis
AP: alkaline phosphatase
ATP: adenosine triphosphate
CXCL1: C‐X‐C motif chemokine ligand 1
CXCL9: C‐X‐C motif chemokine ligand 9
DAPI: 4′,6‐diamidino‐2‐phenylindole
DPH: diphenhydramine
ER: endoplasmic reticulum
ERAD: ER‐associated degradation
FBS: fetal bovine serum
GPCR: G protein‐coupled receptor
HA: hemagglutinin
HaCaT cells: cell line of human keratinocytes
IL‐1β: interleukin‐1β
IL‐6: interleukin‐6
IL‐8: interleukin‐8
NHK cells: normal human keratinocytes
p38 MAPK: p38 MAP kinase
PGK: phosphoglycerate kinase
PROP: 6‐*n*‐propyl‐2‐thiouracil
PTC: phenylthiocarbamide
PTX: pertussis toxin
SRE: serum responsive element
SRF‐RE: serum response factor‐response element
sst‐tag: rat somatostatin type‐3 receptor tag
TAS2R: type‐2 taste receptor
TGFα: transforming growth factor‐alpha
TNFα: tumor necrosis factor‐alpha

## 1 INTRODUCTION

Bitter taste receptors, which are type‐2 taste receptors (TAS2Rs), are chemosensory G protein‐coupled receptors (GPCRs) that protect humans from ingesting toxins.^1^, ^2^, ^3^ The human TAS2R family is composed of 25 members encoded by *TAS2R* genes located on chromosomes 5, 7, and 12.^4^ Each receptor can recognize different exogenous chemical substances.^5^ Recently, TAS2Rs have not only been found in the oral cavity but also throughout the body, such as in the lungs, gastrointestinal tract, kidneys, placenta, male and female sexual organs, and brain^6^, ^7^, ^8^, ^9^, ^10^; however, their physiological roles have not been elucidated. The function of extragustatory TAS2Rs does not lead to taste perception but the recognition of bitter compounds by TAS2Rs evokes distinct tissue‐specific physiological responses and aids host defense against harmful compounds.

The skin provides a first‐line defense against external threats. Recently, TAS2Rs were found to be expressed in the human skin, especially in keratinocytes.^8^, ^11^, ^12^ In fact, Shaw et al. found that TAS2Rs, such as TAS2R3, TAS2R4, TAS2R5, TAS2R8, TAS2R9, TAS2R14, TAS2R30, TAS2R42, and TAS2R60, were expressed in the human skin.^4^ By performing a biopsy study using quantitative polymerase chain reaction (PCR) and a large autopsy experiment using RNA‐seq, these investigators obtained a complete understanding of TAS2R expression. Notably, the functional activities of TAS2Rs in human epidermal keratinocytes have also been reported. TAS2R1 and TAS2R38 are functionally expressed in human epidermal keratinocytes.^11^ In this study, stimulation with diphenidol and amarogentin, the ligands for TAS2R1 and TAS2R38, respectively, induced intracellular Ca^2+^ influx in HaCaT cells, a human keratinocyte cell line. Ho et al. revealed the functional expression of TAS2R14 in human keratinocytes.^12^ These cells responded to α‐thujone, a TAS2R14 agonist, with an increase in intracellular Ca^2+^. These data indicate that TAS2Rs act as chemosensory receptors in epidermal keratinocytes.

In this study, we confirmed that all TAS2Rs are expressed in human keratinocytes and HaCaT cells. We further determined the intracellular localization of TAS2R14 and TAS2R38 using commercially available antibodies. Although TAS2R14 and TAS2R38 belong to the same family of receptors, these receptors have different properties. For example, the expression of TAS2R14 was observed in various tissues, whereas TAS2R38 is hard to detect. Furthermore, TAS2R14 has a wide receptive range, in contrast TAS2R38 has a narrow one. However, in this study we confirmed the similar intracellular localization of these receptors in human keratinocytes. Moreover, using TAS2R38, the best‐characterized bitter taste receptor, we elucidated the intracellular signaling cascade of TAS2R38 in HaCaT cells. TAS2R38 is well‐studied and accounts for human polymorphisms in perceiving the taste of phenylthiocarbamide (PTC) and 6‐*n*‐propyl‐2‐thiouracil (PROP).^13^, ^14^, ^15^

Previous studies have shown that the concentrations of PTC that lead to no taste (neutral) were perceived as extremely bitter by individuals with TAS2R38.^13^, ^14^, ^15^ The taste TAS2R38 protein and the non‐taste form differ in amino acid residues at positions 49, 262, and 296. Functional TAS2R38 contains Pro^49^, Ala^262^, and Val^296^ (PAV‐type), whereas the nonfunctional allele contains Ala^49^, Val^262^, and Ile^296^ (AVI‐type) at these positions. Thus, in our signaling studies, we used the former as a functional receptor and the latter as a nonfunctional receptor against PTC.

We also elucidated the physiological and pathophysiological functions of TAS2Rs in human keratinocytes. Based on our findings, the bitter compounds activated intracellular TAS2Rs, leading to enhanced production of ABCB1 in HaCaT cells and human keratinocytes. The production of ABCB1 was further enhanced in HaCaT cells, which were cultured long‐term (>30 days) with bitter compounds, such as PTC and saccharin. The fluorescent compounds and toxic substances were excreted from these cells but accumulated in parental HaCaT cells. Thus, HaCaT/PTC and HaCaT/saccharin cells had a higher growth rate in the presence of toxic substance than the parental cells. These data suggest that activation of TAS2Rs aids host defense against harmful substances via excretion through ABCB1. Although more extensive studies are needed to gain further insights into the importance of TAS2Rs in keratinocytes, intracellular TAS2Rs is suggested to act as gatekeepers for the first‐line defense against external threats.

## 2 MATERIALS AND METHODS

### 2.1 Resources

Reagents and resources used in this study, their sources, and any associated identifiers are listed in Table S2. Details of their used in this study are in the relevant sections below.

### 2.2 Patients

This study was carried out by the ethical guidelines of the 1975 Declaration of Helsinki. It was approved by the Institutional Research Ethics Committee of the Faculty of Medicine of the University of Tokyo. Informed consent was obtained from the patients for the use of all samples. Skin samples were collected from patients with AD and health controls.

### 2.3 Immunohistochemical detection of TAS2R14 and TAS2R38 in human skin tissues

Tissue sections (5 μm thick) from formaldehyde‐fixed and paraffin‐embedded samples were de‐waxed and rehydrated. In immunohistochemical staining, sections were stained with goat anti‐rabbit TAS2R14 polyclonal IgG, goat anti‐rabbit TAS2R38 polyclonal IgG (Abcam), or goat anti‐rabbit IgG control (Cell Signaling) followed by ABC staining (Vector Laboratories). Diaminobenzidine was used to visualize the staining, and counterstaining with Mayer's hematoxylin was performed.

### 2.4 Construction of TAS2R expression plasmids

The expression plasmids harbor the human TAS2R genes preceded by the first 45 amino acids of rat somatostatin receptor 3 sequence (NH_2_‐MAAVTYPSSVPTTLDPGNASSAWPLDTSLGNASAGTSLAGLAVSG‐COOH) (5′‐ATGGCCGCTGTTACCTATCCTTCATCCGTGCCTACGACCTTGGACCCTGGGAATGCATCCTCAGCCTGGCCCCTGGACACGTCCCTGGGGAATGCATCTGCTGGCACTAGCCTGGCAGGACTGGCTGTCAGTGGC‐3′).^5^, ^16^ N‐terminally HA‐tagged constructs (HA‐sst‐TAS2Rs) was prepared as described previously.^17^, ^18^

### 2.5 Cell culture and transfection

HeLa, HEK293T, HEK293A, G_αi_‐deficient HEK293A,^18^ G_α12/13_‐deficient HEK293A,^19^ and HaCaT^20^ cells were cultured in Dulbecco's modified Eagle's medium (DMEM, Nacalai Tesque) supplemented with 10% fetal bovine serum. These cells were transfected with a plasmid harboring TAS2Rs using Lipofectamine 2000 (Invitrogen) according to the manufacturer's protocol. Normal human epidermal keratinocytes (derived from darkly pigmented newborn foreskins, passage 3; Cascade Biologics) were cultured in medium 154 (Cascade Biologics, 0.2 mM Ca^2+^) supplemented with a commercial cocktail of growth factors (HKGS, consisting of 0.2 ng/mL human recombinant EGF, 0.18 mg/mL hydrocortisone, 5 mg/mL insulin, 5 mg/mL transferrin, and 0.2% (v/v) bovine pituitary extract) and an antibiotic/antimycotic solution.

### 2.6 Isolation of total RNA, RT‐PCR analysis

Total RNA was isolated using an RNeasy Mini Kit (Qiagen). cDNA synthesis was carried out by SuperScript III reverse transcriptase (Invitrogen) using 1 μg total RNA as a template. RT‐PCR was performed by KOD‐plus neo polymerase. The primers used in these experiments were listed in Table S3.^21^ β‐actin was used as a control gene.

### 2.7 Real‐time quantitative PCR analysis

Real‐time quantitative PCR was performed with a Thunderbird SYBR Green (TOYOBO). The data were normalized to β‐actin amplified by PCR. The primers used in these experiments are listed in Table S4.

### 2.8 Calcium mobilization assay

Transiently transfected HeLa cells were loaded with 3 μM Fura‐2 AM (Dojindo) in a modified Hepes‐Tyrode's BSA buffer [25 mM HEPES‐NaOH (pH 7.4), 140 mM NaCl, 2.7 mM KCl, 1.0 mM CaCl_2_, 12 mM NaHCO_3_, 5.6 mM D‐glucose, 0.37 mM NaH_2_PO_4_, 0.49 mM MgCl_2_, 0.1% w/v BSA], containing 0.01% pluronic acid (Molecular Probes) at 37°C for 1 h. The cells were washed and resuspended in HEPES‐Tyrode's BSA buffer at a density of 1 × 10^6^ cells/mL. The cell suspension (0.5 mL) was applied to a CAF‐110 system (JASCO Corp.), and 5 μL of ligand solution was added. The ligands were dissolved in HEPES‐Tyrode's BSA buffer. The intracellular Ca^2+^ concentration was measured by determining the ratio of emission fluorescence of 500 nm by excitation at 340 and 380 nm. The free Ca^2+^ concentration was calculated from the equation: Ca^2+^_i_ = *K*_d_[(*F − F*_min_)/(*F*_max_ *− F*)], where *K*_d_ represents the Ca^2+^ binding dissociation constant (224 nM for Fura‐2), *F* is the 500‐nm fluorescence ratio, *F*_max_ is the maximal fluorescence ratio determined after addition of 0.1% Triton X‐100 to permeabilize the cells in the presence of 1 mM Ca^2+^, and *F*_min_ is the minimal fluorescence ratio determined after permeabilization and addition of 5 mM EGTA.

### 2.9 Preparation of cell membrane fractions

The cells were washed with ice‐cold Tris buffer [20 mM Tris–HCl (pH 7.4), 0.3 M sucrose, 1 mM sodium orthovanadate], and collected in HEPES buffer [25 mM HEPES‐NaOH (pH 7.4), 10 mM MgCl_2_, 0.3 M sucrose, 1 mM sodium orthovanadate, supplemented with cOmplete™ protease inhibitor cocktail]. Subsequently, the cells were sonicated for 20 min using a Bioruptor (CosmoBio) and centrifuged at 8000 × *g* for 10 min at 4°C to remove cellular debris. The resulting supernatant was ultracentrifuged at 100,000 × *g* for 1 h at 4°C. The microsome pellet was resuspended in a buffer containing the above‐mentioned HEPES buffer. The samples were then subjected to SDS‐PAGE as cell membrane fractions.

### 2.10 Immunofluorescence confocal microscopy analysis

Cells (5 × 10^5^ cells) were seeded into collagen‐coated glass‐bottomed 35‐mm dish. After transfection and incubation for 48 h, the cells were fixed with 2% paraformaldehyde for 10 min at room temperature and rinsed twice with ice‐cold PBS. Subsequently, the cells were incubated with 1/4 × permeabilization reagent for 10 min at room temperature. Then, primary antibodies (anti‐HA [3F‐10] and anti‐organelle marker) were added, and the mixture was incubated for 2 h. After the cells were washed with ice‐cold PBS, secondary antibodies (Alexa Fluor 488‐conjyugated anti‐rat IgG and Alexa Fluor 546‐conjyugated anti‐mouse IgG) were added, and incubation was continued for 2 h. 4′,6‐diamidino‐2‐phenylindole was used to stain for the nucleus. Images were obtained using All‐In One Confocal Microscopy System (Keyence).

### 2.11 Flow cytometry

For staining, cells were incubated with the antibody against HA‐tag (3F‐10) in PBS containing 2% goat serum at room temperature for 30 min, followed by staining with PE‐conjugated antibody recognizing rat IgG at room temperature for 30 min. The Cube6 (Sysmex) was used for flow cytometry.

### 2.12 Reporter gene assay

For the analyses of TAS2R38, HEK293T cells were plated in a 24‐well plate (2 × 10^5^ cells/well) and transfected with 0.4 μg per well of plasmid DNA using Lipofectamine 2000. The ratio of transfected plasmid was kept constant at 4:2.5:1 for SRE‐NanoLuc‐pNL (NLucP/SRE/Hygro), SRF‐RE‐NanoLuc‐pNL (NLucP/SRF‐RE/Hygro), CRE‐NanoLuc‐pNL (NLucP/CRE/Hygro), or NFAT‐RE‐NanoLuc‐pNL (NLucP/NFAT‐RE/Hygro): HA‐sst‐TAS2R38/pcDNA3: phosphoglycerate kinase (PGK)‐firefly luciferase‐pGL4.53 (luc2/PGK). The cells were starved for 6 h and stimulated with 1 mM PTC for 15 h. In the taste cells, TAS2Rs receives their specific ligands on the cell surface. In contrast, these receptors are localized intracellularly in keratinocytes. In these cells, because the ligands were pass through the cellular membrane to bind the cognate receptors, higher doses of ligands were required to activate TAS2Rs in our study. The luciferase assay was performed using the Nano‐Glo Dual‐Luciferase Reporter Assay System (Promega) and the GloMax 20/20 luminometer. NanoLuc activity was normalized to the levels of firefly luciferase activity.

### 2.13 TGFα shedding assay

The transforming growth factor‐alpha (TGFα) shedding assay was carried out as previously described.^22^ In brief, HEK293T cells were seeded in 12‐well plates (2 × 10^5^ cells/well), 24 h prior to transfection. The cells were transfected with plasmids encoding alkaline phosphatase (AP)‐TGFα (0.25 μg/well), HA‐sst‐TAS2R38 or dopamine D2 receptor (0.05 μg/well), and G_α_ mixture, G_αq_, G_αq/i1_, G_αq/i3_, G_αq/12_, G_αq/13_, and G_αq/s_ (0.05 μg/well). After 24 h, the cells were collected and washed with PBS, and then suspended in Hank's balanced salt solution (HBSS) solution containing 5 mM HEPES (pH 7.4). The cell suspension was plated in a 96‐well plate (80 μL/well), stimulated with 20 μL of HBSS solution containing 5 mM PTC or 50 nM dopamine hydrochrolide, 3,4‐dihydroxyphenethylamine hydrochloride for 1 h at 37°C. The 96‐well plates were centrifuged for 2 min at 200 × *g*, and 80 μL of the supernatant was transferred into another 96‐well plate. An 80‐μL portion of 2× pNPP buffer [10 mM pNPP in 40 mM Tris–HCl (pH 9.5), 40 mM NaCl, and 10 mM MgCl_2_] was added to both the supernatant and the cells. The absorbance at 405 nm (OD_405_) of both plates was read before and after a 1‐h incubation at 37°C, using a microplate reader (Thermo Fisher Scientific). AP‐TGFα release was calculated by the following formulae:

$\mathrm{AP}-\text{TGFα in supernatant}\;\left(\%\right)=\left({\mathrm{\Delta OD}}_{405}\mathrm{Sup}/\left[{\mathrm{\Delta OD}}_{405}\mathrm{Sup}+{\mathrm{\Delta OD}}_{405}\text{Cell}\right]\right)\times 100.$

$\mathrm{AP}-\text{TGFα release}\;\left(\%\right)=\mathrm{AP}-\text{TGFα in supernatant under}\;a\;\text{stimulated condition}\;\left(\%\right).$


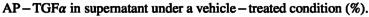


### 2.14 Rhodamine 123 efflux assay

Cells (2 × 10^5^ cells) were seeded into collagen‐coated glass‐bottomed 35 mm dish. Intracellular retentions of rhodamine 123 were examined by incubation of 10 μM rhodamine 123 for 1 h in darkness at 37°C in the presence or absence of 20 μM verapamil. After the cell were washed for three times with cold PBS, images were acquired by fluorescence microscopy, 488 nm excitation and 535 nm emission wavelength.^23^ Fluorescence intensities were determined by using Image J software.

### 2.15 Cell toxicity assay

Cell toxicity of diphenhydramin (DPH) was determined using Zombie Red Fixable Viability Kit (BioLegend) according to the manufacturer's protocol.

### 2.16 Statistical analysis

For each experiment, at least three independent experiments were performed. The images obtained from one representative experiment were presented. To determine statistical significance, values were compared by Student's *t*‐test, or two‐way ANOVA with Tukey post hoc test using Prism 7 software. The differences were considered significant if *p* values were less than 0.05.

## 3 RESULTS

### 3.1 TAS2Rs in human keratinocytes

**FIGURE 1:**
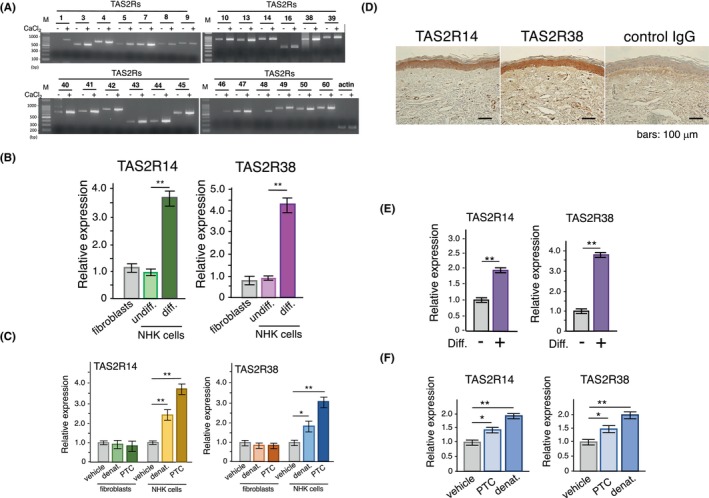
TAS2Rs in human keratinocytes. (A) Expression of human TAS2Rs in NHK cells. NHK cells were differentiated via culture with 1 mM CaCl_2_ for 3 days. (B) Increased expression of TAS2R14 and TAS2R38 during NHK cell differentiation *n* = 5. (C) Increased expression of TAS2R14 and TAS2R38 following stimulation of differentiated NHK cells with 1 mM PTC and 1 mM denatonium *n* = 4. (D) Representative images of immunohistochemical staining for TAS2R14 and TAS2R38 in lesioned skin from healthy donor. Scale bar, 100 μm. (E) Increased expression of TAS2R14 and TAS2R38 during HaCaT cell differentiation *n* = 4. (F) Increased expression of TAS2R14 and TAS2R38 in differentiated HaCaT cells following stimulation with 1 mM PTC or 1 mM denatonium in differentiated HaCaT cells *n* = 4. Data are expressed as mean ± SEM. *p < 0.05, **p < 0.01 using two‐way ANOVA followed by Tukey's post hoc test (B, C, F) and **p < 0.01 using Student's *t*‐test (E).

First, we confirmed the expression of 25 human TAS2Rs in normal human keratinocytes (NHK cells) using RT‐PCR (Figure 1A). The expression of these receptors was further enhanced during NHK differentiation when cultivated in the presence of 1.5 mM CaCl_2_ (Figure 1A). For example, the expression of TAS2R14 and TAS2R38 increased by approximately 3.5–4.5‐fold during differentiation (Figure 1B). In these differentiated cells, the expression levels of TAS2R14 and TAS2R38 were further augmented by stimulation with bitter compounds such as PTC and denatonium (Figure 1C). We confirmed the production of TAS2R14 and TAS2R38 proteins in the human skin using commercially available specific antibodies. As shown in Figure 1D, the production of TAS2R14 and TAS2R38 was detected in the human cornified layer of tissues from healthy skin. In addition, all TAS2Rs were found to be expressed in the immortalized human keratinocyte cell line, HaCaT (Figure S1). As observed in NHK cells, the expression levels of TAS2R14 and TAS2R38 were enhanced during HaCaT differentiation in the presence of 1.5 mM CaCl_2_ (Figure 1E) and augmented by exposure to PTC or denatonium (Figure 1F). Taken together, these results indicate the existence of bitter taste receptors on human skin. In this study, we used HaCaT cells as a keratinocyte model to elucidate the functional significance of these receptors on the skin.

### 3.2 Intracellular localization of TAS2R14 and TAS2R38 in keratinocytes

**FIGURE 2:**
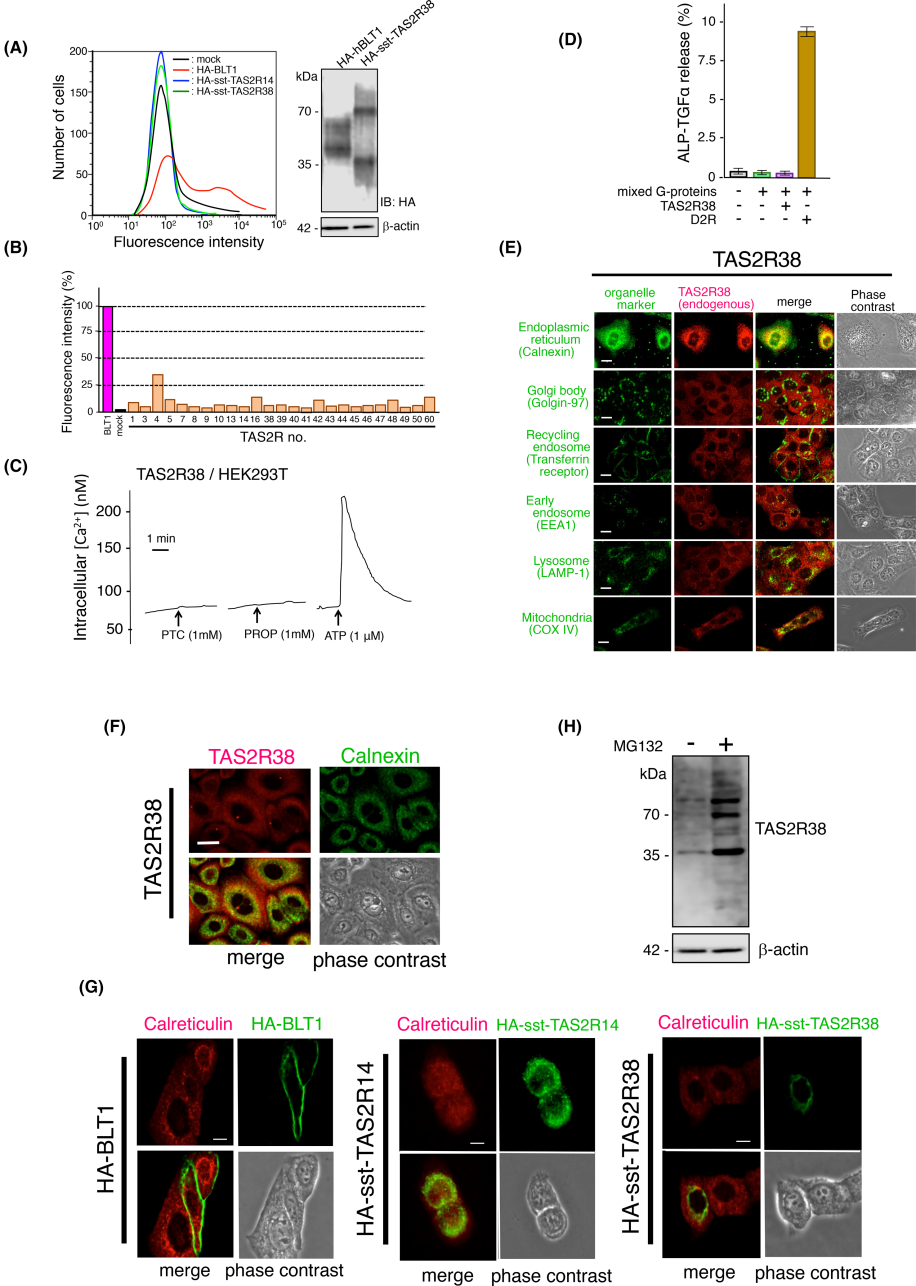
Intracellular localization of TAS2R14 and TAS2R38 in keratinocytes. (A) Expression of HA‐sst‐tagged TAS2R14 and HA‐sst‐tagged TAS2R38 in HEK293T cells. The HEK293T cells were transfected with the indicated receptors. Cells were stained with a primary HA antibody, followed by a PE‐conjugated secondary antibody, and HA‐positive cells were detected using flow cytometry (left). In this experiment, HA‐BLT1 located on the plasma membrane was used as a control. The production of receptor proteins was confirmed using SDS‐PAGE followed by immunoblotting (right). (B) Expression of 25 human TAS2Rs in HEK293T cells. HEK293T cells were transfected with the HA‐sst‐tagged receptors. Cells were stained with a primary HA antibody, followed by a PE‐conjugated secondary antibody, and HA‐positive cells were detected using flow cytometry. HA‐BLT1 and empty vector (pcDNA3) were used as positive and negative controls, respectively. The fluorescence intensity of transfected cells was measured. (C) Intracellular [Ca^2+^] responses in HEK293T cells expressing HA‐sst‐TAS2R38 elicited by 1 mM PTC and 1 mM PROP. The response to 1 μM ATP was included as a positive control. (D) TGFα shedding assay demonstrating cell surface receptor activity.^22^ HEK293T cells were transfected with AP‐TGFα, HA‐sst‐TAS2R38, or the dopamine D2 receptor and G_α_ mixture, G_αq_, G_αq/i1_, G_αq/i3_, G_αq/12_, G_αq/13_, and G_αq/s_. After stimulation of HA‐sst‐TAS2R38 expressing cells with 1 mM PTC, the release of AP‐TGFα into the culture medium was determined. The dopamine D2 receptor was stimulated with 10 nM dopamine hydrochloride and 3,4‐dihydroxyphenethylamine hydrochloride. (E) Immunofluorescence confocal microscopic analysis of TAS2R38 with various organelle marker proteins. The HaCaT cells were subjected to immunocytochemical analysis. Organelle marker proteins (green) were detected using anti‐calnexin (ER marker), anti‐golgin‐97 (Golgi body marker), anti‐transferrin receptor (recycling endosome marker), anti‐EEA1 (early endosome marker), anti‐LAMP‐1 (lysosomal marker), and anti‐COX IV (mitochondrial marker). Endogenous TAS2R38 expression was detected using anti‐TAS2R38 (red). Scale bar, 10 μm. (F) Immunofluorescence confocal microscopic analysis of endogenous TAS2R38 in NHK cells. TAS2R38 expression was detected using anti‐TAS2R38 (red). Calnexin was detected as an ER marker using an anti‐calnexin antibody (green). Scale bar, 10 μm. (G) Immunofluorescence confocal microscopic analysis of HA‐sst‐tagged TAS2R14 and HA‐sst‐tagged TAS2R38 in HaCaT cells. HaCaT cells were transfected with HA‐sst‐TAS2R14, HA‐sst‐TAS2R38, or HA‐BLT1, and subjected to immunocytochemical analysis. HA‐BLT1 (left), HA‐TAS2R14 (middle), and HA‐TAS2R38 (right) were detected using anti‐HA (green). HA‐BLT1 was used as the control receptor on the cell surface. Calreticulin was detected using anti‐calreticulin (red). Scale bar, 10 μm. (H) Western blot showing HA‐sst‐TAS2R38 in membrane fractions from HEK293T cells expressing HA‐sst‐TAS2R38 treated with or without 20 μM MG132. β‐Actin was employed as an experimental and loading control.

Previous studies revealed that the production of TAS2R proteins was augmented by the fusion of the rat somatostatin type‐3 receptor N‐terminus (sst‐tag) to the N terminal of TAS2Rs (HA‐sst‐TAS2Rs), which might be due to the facilitation of proper folding (Figure S2A).^5^, ^24^ We confirmed the enhanced generation of HA‐sst‐TAS2R14 and HA‐sst‐TAS2R38 proteins compared to HA‐TAS2R14 and HA‐TAS2R38 in HEK293T cells, which do not endogenously express TAS2Rs (Figure S2B). However, in HA‐sst‐TAS2R14‐ and HA‐sst‐TAS2R38‐expressing cells, the surface expression of these receptors was significantly lower than that of the leukotriene B_4_ type‐1 receptor (BLT1), a typical plasma membrane receptor (Figure 2A). As the amount of HA‐sst‐TAS2R38 protein produced in these cells was not significantly different from that of HA‐BLT1, we predicted that HA‐sst‐TAS2Rs could accumulate intracellularly. Reduced surface expression of the HA‐sst‐tagged receptors was further confirmed in cells expressing other TAS2Rs (Figure 2B). The lack of functional HA‐sst‐TAS2R38 on the cell surface was revealed by its response to PTC and PROP, known agonists of this receptor.^5^ As shown in Figure 2C, although HEK293T cells exhibited an intracellular Ca^2+^ increase upon stimulation with adenosine triphosphate (ATP) via the intrinsic ATP receptor, we did not investigate the apparent response of PTC and PROP in HA‐sst‐TAS2R38 expressing cells, supporting the lack of functional receptors on the cell surface. Furthermore, based on the TGFα‐shedding assay,^22^ AP‐TGFα release by PTC, which occurs via the activation of the mixed G_α_‐chimera proteins, G_αq_, G_αq/12_, G_αq/13_, G_αq/s_, and G_αq/i1_, did not occur with the expression of HA‐sst‐TAS2R38. However, activation of G_αq/i1_ via the dopamine type‐4 receptor resulted in dopamine‐induced AP‐TGFα release into the culture medium (Figure 2D). To gain further insights into the importance of TAS2Rs in keratinocytes, we determined the intracellular localization of these receptors in HaCaT cells using immunofluorescence confocal microscopy. By using commercially available specific antibodies against human TAS2R14 and TAS2R38, the localization of these endogenous receptors in HaCaT cells was determined via co‐staining with several organelle markers, such as calnexin (endoplasmic reticulum, ER), golgin‐97 (Golgi apparatus), transferrin receptor (recycling endosome), early endosome antigen 1 (EEA1; early endosome), lysosomal‐associated membrane protein 1 (LAMP‐1; lysosome), and cytochrome *c* oxidase (COX IV; mitochondria). As shown in Figure 2E and Figure S2C, both endogenous TAS2R14 and TAS2R38 were confirmed to accumulate intracellularly in HaCaT cells and presumably colocalized with calnexin. Similar colocalization of TAS2R14 and TAS2R38 with calnexin was confirmed in NHK cells (Figure 2F and Figure S2D). In addition, we co‐stained endogenous TAS2R14 and TAS2R38 with ERseeing, an ER‐staining dye comprising a rhodol‐type green fluorescent dye and a thioester‐type protein‐labeling group, in which the rhodol derivative has a high affinity for the ER membrane, and investigated the retention of both receptors in the ER (Figure S2E). The localization of both receptors in the ER was confirmed using an anti‐HA‐tag antibody in HaCaT cells producing HA‐sst‐TAS2R14 or HA‐sst‐TAS2R38 (Figure 2G). As the treatment of HA‐sst‐TAS2R38‐expressing cells with MG132 led to the augmentation of this receptor, an excess of this receptor in the ER could be degraded via the ER‐associated degradation system (Figure 2H). Collectively, these data suggest the intracellular localization of TAS2R14 and TAS2R38 in HaCaT cells, presumably in the ER.

### 3.3 Coupling of intracellular TAS2R38 with G_α12/13_

**FIGURE 3:**
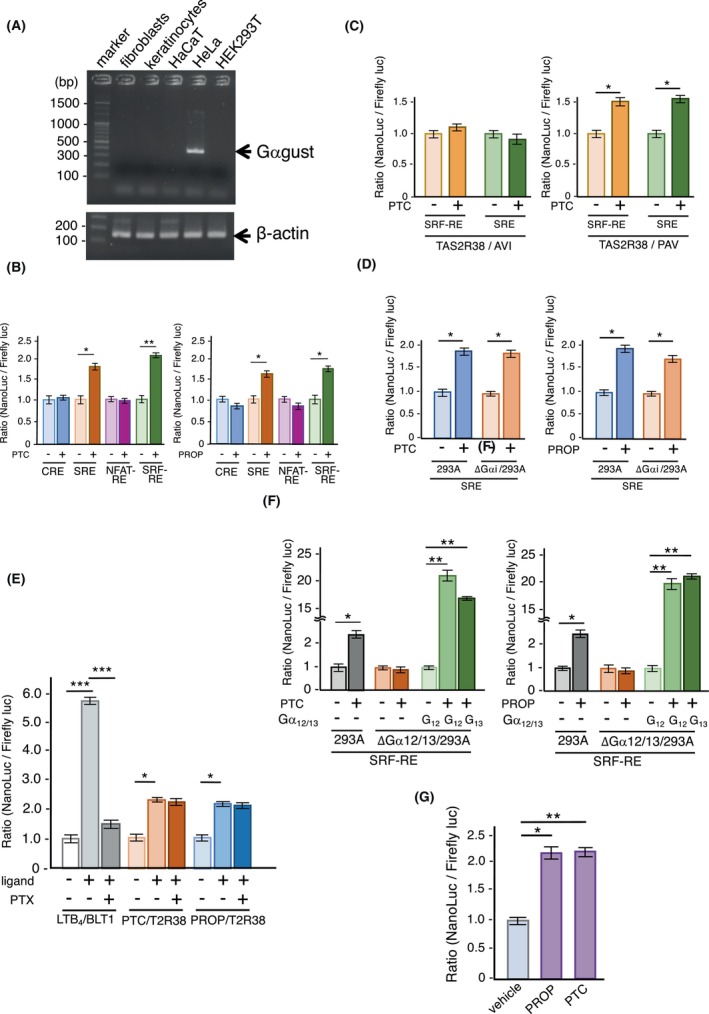
Coupling of intracellular TAS2R38 with G_α12/13_. (A) Expression analysis of G_αgust_ in human fibroblasts, keratinocytes, HaCaT, HeLa, and HEK293T cells. Total RNA was extracted from these cells and RT‐PCR of G_αgust_ was performed using these cDNAs as templates. (B) Activation of SRE and SRF‐RE via TAS2R38 by stimulation with 1 mM PTC (left) and 1 mM PROP (right). In TAS2R38 expressing HEK293T cells, NanoLuc luciferase activity driven by SRF‐RE, NFAT‐RE, CRE, or SRE was quantified as the ratio of NanoLuc/Firefly luciferase activity *n* = 4. (C) Activation of SRE and SRF‐RE by TAS2R38/PAV (right) but not TAS2R38/AVI (left) following stimulation with 1 mM PTC in HEK293T cells *n* = 4. (D) Effect of G_αi_ deficiency on the activation of SRE stimulated with 1 mM PTC and 1 mM PROP in HEK293A cells *n* = 4. (E) Effect of PTX on the activation of SRE stimulated with 1 mM PTC and 1 mM PROP in HEK293A cells. Reduced activity induced by PTX via the leukotriene B_4_/BLT1/G_αi_ axis was used as the control *n* = 4. (F) Effect of G_α12/13_ deficiency on activation of SRF‐REs stimulated with 1 mM PTC and 1 mM PROP in HEK293A cells *n* = 4. (G) Activation of SRF‐RE by stimulating HaCaT cells with 1 mM PTC and 1 mM PROP in HaCaT cells. HaCaT cells were transfected with SRF‐RE‐fused NanoLuc luciferase, and luciferase activity driven by SRF‐RE was quantified as the ratio of NanoLuc/firefly luciferase activity *n* = 4. Data are expressed as mean ± SEM. *p < 0.05, **p < 0.01, ***p < 0.001 using two‐way ANOVA followed by Tukey's post hoc test (B–G).

In taste cells, TAS2Rs preferentially couple with G_αgust_, a taste cell‐specific G protein.^25^ However, the expression of G_αgust_ was not detected in HaCaT and NHK cells, which produce TAS2Rs, suggesting the involvement of other G‐proteins in TAS2R signaling in these cells (Figure 3A). Thus, we explored the signaling pathways of TAS2Rs in HEK293T cells using a NanoLuc reporter system (Figure S3A).^17^ As shown in Figure 3B, stimulation with PTC and PROP led to significant increases in the NanoLuc activities of HA‐sst‐TAS2R38‐expressing HEK293T cells co‐expressed with a serum responsive element (SRE) or serum response factor‐response element (SRF‐RE)‐fused reporter. Thus, TAS2R38 may couple with G_αi_ and/or G_α12/13_ proteins (Figure S3A). According to previous reports, at least two types of polymorphisms are associated with TAS2R38: Pro^49^/Ala^262^/Val^296^ (TAS2R38/PAV) and Ala^49^/Val^262^/Ila^296^ (TAS2R38/AVI); the former polymorphism is activated by PTC, whereas the latter is not (Figure S3B).^26^ In reporter experiments, SRF‐RE‐ and SRE‐dependent NanoLuc activity was evoked by stimulation with PTC in TAS2R38/PAV‐expressing HEK393T cells; however, TAS2R38/AVI‐expressing cells did not exhibit these responses (Figure 3C). Hence, the reporter activity observed in these experiments was induced by exogenously expressed TAS2R38/PAV after stimulation with specific ligands. As shown in Figure 3B, the coupling of TAS2R38 with G_αi_ and/or G_α12/13_ proteins was predicted. However, the deficiency of G_αi_ in HEK293A cells did not affect PTC or PROP‐induced activation of the SRE via TAS2R38/PAV (Figure 3D). In addition, the TAS2R38/PAV‐SRE pathway was not inhibited by pretreatment with 100 ng/mL of pertussis toxin (PTX), suggesting G_αi_‐independent signaling (Figure 3E). In contrast, in G_α12/13_‐null cells, the reduced activity of the SRF‐RE via TAS2R38/PAV was recovered by the co‐expression of G_α12_ or G_α13_, suggesting the involvement of G_α12/13_ in TAS2R38/PAV‐eliciting reporter activity (Figure 3F). We further examined PTC or PROP‐induced activation of the SRF‐RE via endogenous TAS2R38 in HaCaT cells. Prior to this experiment, we determined the type of TAS2R38 polymorphism in several human cell lines, including HaCaT cells, using PCR with genomic DNA as the template (Table S4). Based on our findings, HaCaT cells harbor the PAV but not the AVI form of TAS2R38. SRF‐RE was significantly activated in HaCaT cells following stimulation with PTC and PROP (Figure 3G), implying the involvement of G_α12_ and G_α13_ in signaling via TAS2R38 in keratinocytes.

### 3.4 Activation of the p38 MAP kinase/NF‐κB axis

**FIGURE 4:**
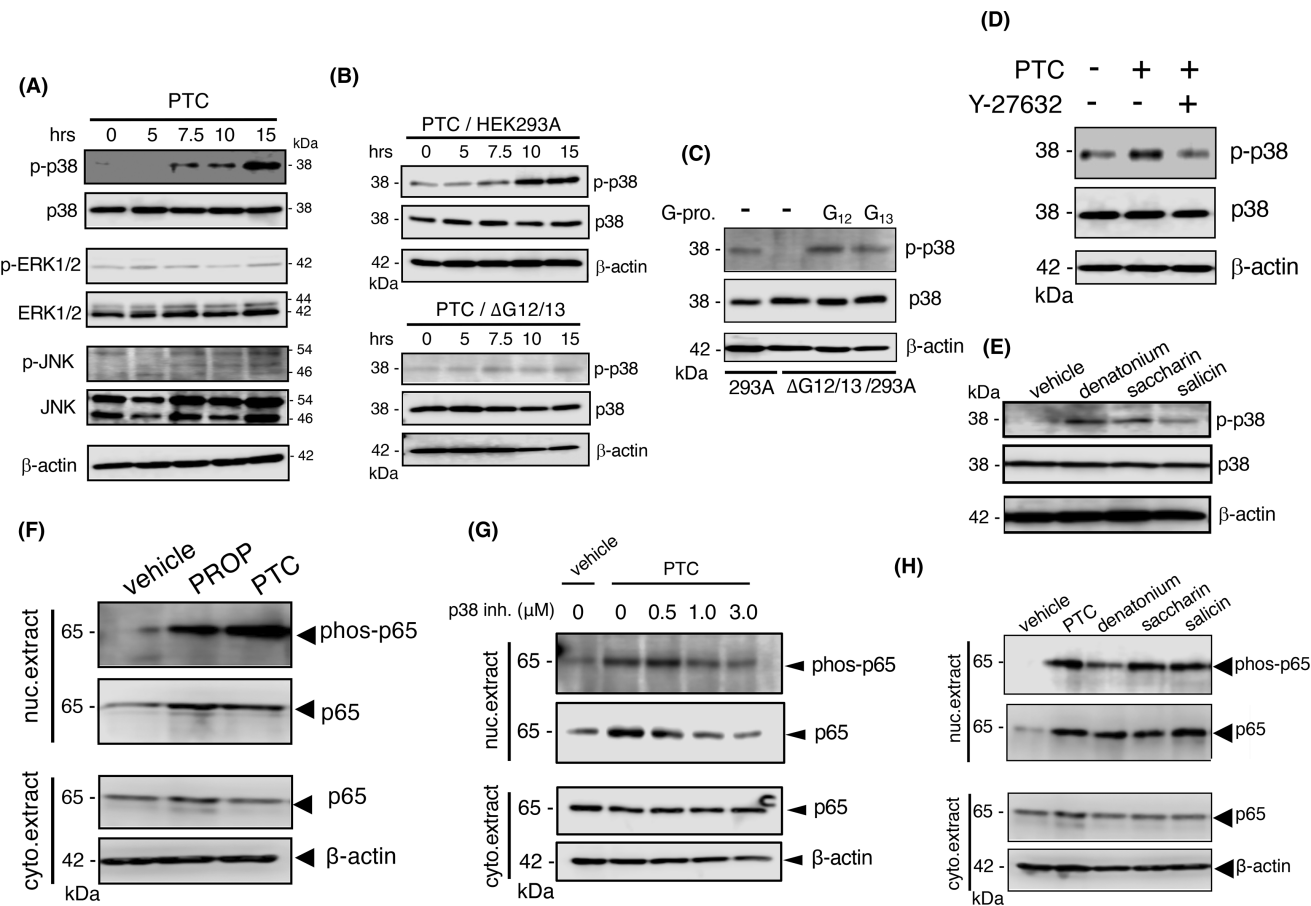
Activation of the p38 MAPK/NF‐κB axis. (A) Western blot of p38 MAPK, phosphorylated p38 MAPK (p‐p38 MAPK), ERK1/2, phosphorylated ERK1/2 (p‐ERK1/2), JNK, and phosphorylated JNK (p‐JNK) in lysates from HEK293T cells expressing HA‐sst‐TAS2R38 at the indicated time points after stimulation with 1 mM PTC. (B) Western blot of p38 MAPK and p‐p38 MAPK in lysates from HEK293A and G_α12/13_‐null (ΔG_α12/13_) HEK293A cells expressing HA‐sst‐TAS2R38 at the indicated time points after stimulation with 1 mM PTC. (C) Western blot of p38 MAPK and p‐p38 MAPK in lysates from HEK293A and ΔG_α12/13_ HEK293A cells expressing HA‐sst‐TAS2R38 with G_α12_ or G_α13_ at 15 h after 1 mM PTC stimulation. (D) Western blot of p38 MAPK and p‐p38 MAPK in lysates from HEK293A cells expressing HA‐sst‐TAS2R38 treated with or without 10 μM Y27632 after 1 mM PTC stimulation for 15 h. (E) Western blot of p38 MAPK and p‐p38 MAPK in HaCaT cell lysates after stimulation with 1 mM denatonium, 1 mM saccharin, or 1 mM salicin for 15 h. (F) Western blot of the p65 subunit of NF‐κB and phosphorylated p65 (phos‐p65) in cytoplasmic and nuclear extracts from HEK293T cells expressing HA‐sst‐TAS2R38 after stimulation with 1 mM PTC or 1 mM PROP for 15 h. (G) Western blot of p65 and phos‐p65 in the cytoplasmic and nuclear extracts from HEK293T cells expressing HA‐sst‐TAS2R38 treated with or without SB203580 at the indicated concentrations prior to stimulation with 1 mM PTC for 15 h. (H) Western blot of p65 and phos‐p65 in the cytoplasmic and nuclear extracts from differentiated HaCaT cells stimulated with 1 mM denatonium, 1 mM saccharin, or 1 mM salicin for 15 h. β‐Actin was employed as an experimental and loading control.

We examined the signaling pathway via TAS2Rs, particularly the involvement of various MAP kinases elicited by intracellular receptors. As shown in Figure 4A and Figure S4A, p38 MAP kinase (p38 MAPK) was activated 7.5–10 h after the addition of PTC or PROP to HaCaT cells; however, ERK1/2 and JNK1/2/3 were not phosphorylated under these conditions. The activation of p38 MAPK was further investigated in TAS2R38/PAV‐expressing HEK293T cells, but not in TAS2R38/AVI‐expressing cells, by the stimulation with PTC (Figure S4B). Similar activation of p38 MAPK was observed in NHK cells (Figure S4C), suggesting that this kinase is involved in signaling via intracellular TAS2Rs. The significance of G_α12/13_ in the activation of p38 MAPK was confirmed in HA‐sst‐TAS2R38/PAV‐expressing HEK293A cells lacking both G_α12_ and G_α13_ (Figure 4B). The defect in p38 MAPK activation elicited by PTC was recovered by the exogenous expression of G_α12_ or G_α13_ in these cells (Figure 4C). The activation of p38 MAPK by PTC stimulation was reduced by treatment with Y‐27632, a ROCK inhibitor (Figure 4D and Figure S4D), suggesting the involvement of RhoA/ROCK in the activation of p38 MAPK. Moreover, we confirmed the activation of p38 MAPK by stimulation with other bitter compounds such as denatonium, saccharin, and salicin, in HaCaT cells (Figure 4E). These data indicate signaling of intracellular TAS2Rs via the G_α12/13_/RhoA/ROCK/p38 MAPK pathway. According to several studies, the potentiation of p38 MAPK activity is required for the action of NF‐κB.^27^, ^28^ Thus, we examined the activation of NF‐κB, namely translocation of the phosphorylated NF‐κB, via stimulation with PTC or PROP in both HaCaT and NHK cells. As shown in Figure 4F, the phosphorylation of p65, a subunit of NF‐κB, was detected in PTC or PROP‐stimulated HaCaT cells. Similar results were obtained in NHK cells (Figure S4E), suggesting the activation of NF‐κB by the bitter compounds. Consistent with this finding, PTC‐induced NF‐κB activation was found to be inhibited by SB203580, an inhibitor of p38 MAPK (Figure 4G). NF‐κB was activated by other bitter substrates, such as denatonium, saccharin, and salicin, in HaCaT (Figure 4H) and NHK cells (Figure S4F), implicating the p38 MAPK/NF‐κB axis in the signaling evoked by bitter compounds, possibly via intracellular TAS2Rs in keratinocytes.

### 3.5 Enhanced expression of ABC‐transporters via intracellular TAS2R activation

**FIGURE 5:**
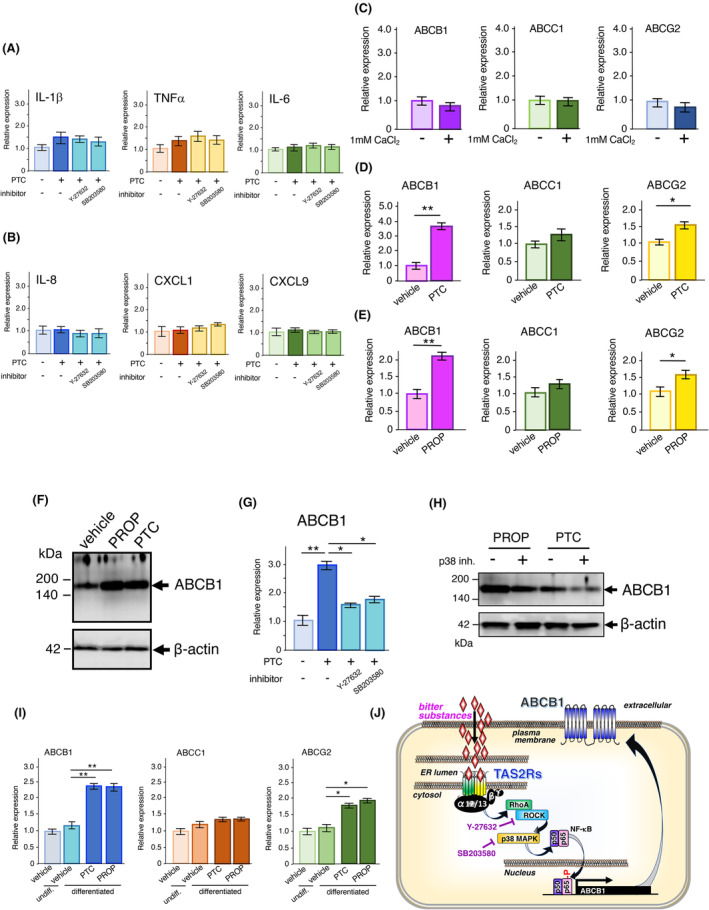
Expression of proinflammatory cytokines, chemokines, and ABC transporters in HaCaT cells stimulated with PTC. (A) Expression of IL‐1β, TNF‐α, and IL‐6 in PTC‐treated HaCaT cells. HaCaT cells were treated with 1 mM PTC for 15 h in the presence or absence of a ROCK blocker (Y‐27632) or p38 MAPK‐inhibitor (SB203580), and total RNAs was extracted. Real‐time PCR of the cytokines was performed using cDNAs as templates *n* = 4. (B) Expression of IL‐8, CXCL1, and CXCL9 in PTC‐treated HaCaT cells. HaCaT cells were treated with 1 mM PTC for 15 h in the presence or absence of a ROCK‐blocker or p38 MAPK‐inhibitor, and total RNA was extracted. Real‐time PCR analysis of the chemokines was performed using the cDNAs as templates *n* = 4. (C) Expression of ABCB1, ABCC1, and ABCG2 in differentiated HaCaT cells. HaCaT cells were differentiated using 1 mM CaCl_2_ for 3 days. On Days 0 and 3, total RNAs were extracted. Real‐time PCR analysis of ABCB1, ABCC1, and ABCG2 was performed using the cDNAs as templates *n* = 4. (D) Expression of ABCB1, ABCC1, and ABCG2 in differentiated HaCaT cells stimulated with or without 1 mM PTC for 15 h. Real‐time PCR analysis of ABCB1, ABCC1, and ABCG2 was performed using the cDNAs as templates *n* = 4. (E) Expression of ABCB1, ABCC1, and ABCG2 in differentiated HaCaT cells treated with or without 1 mM PROP for 15 h. Real‐time PCR analysis of ABCB1, ABCC1, and ABCG2 was performed using the cDNAs as templates *n* = 4. (F) Western blotting of ABCB1 in the membrane fractions from differentiated HaCaT cells stimulated with 1 mM PTC or 1 mM PROP for 15 h. β‐Actin was employed as an experimental and loading control. (G) Expression of ABCB1 in PTC‐treated HaCaT cells. HaCaT cells were treated with 1 mM PTC for 15 h in the presence or absence of a ROCK‐blocker or a p38 MAPK‐inhibitor, and total RNAs were extracted. Real‐time PCR analysis of ABCB1 was performed using cDNAs as templates *n* = 4. (H) Involvement of p38 MAPK in the induction of ABCB1 production in HaCaT cells following stimulation with 1 mM PTC or 1 mM PROP. β‐Actin was employed as an experimental and loading control. (I) Expression of ABCB1, ABCC1, and ABCG2 in differentiated NHK cells. NHK cells were differentiated using 1 mM CaCl_2_ for 3 days. On Day 3, the cells were stimulated with 1 mM PTC or 1 mM PROP for 15 h and total RNA was extracted. Real‐time PCR analysis of ABCB1, ABCC1, and ABCG2 was performed using the cDNAs as templates *n* = 4. Data are expressed as mean ± SEM. *p < 0.05, **p < 0.01 using two‐way ANOVA followed by Tukey's post hoc test (A, B, G, I). *p < 0.05, **p < 0.01 using Student's *t*‐test (C–E). (J) Schematic model of the signaling pathway via TAS2Rs. After stimulation of TAS2Rs by bitter compounds, the G_α12/13_/RhoA/ROCK/p38 MAPK/NF‐κB pathway is activated, and the expression levels of several ABC transporters, including ABCB1, are increased.

According to previous studies, various proinflammatory cytokines, chemokines,^29^, ^30^, ^31^ and ABC‐transporters^32^, ^33^, ^34^, ^35^, ^36^ are produced in keratinocytes. Thus, we examined the expression of several pro‐inflammatory cytokines and chemokines, such as IL‐1β, TNF‐α, IL‐6, IL‐8, CXCL1, and CXCL9, in HaCaT cells stimulated with PTC. Notably, exposure to 1 mM PTC for 15 h did not increase the mRNA levels of these cytokines and chemokines (Figure 5A,B). As various harmful substances are excreted by several ABC transporters, such as ABCB1, ABCC1, and ABCG2,^37^ we examined the effects of bitter compounds on the expression of ABC transporters in HaCaT cells. The expression of ABCB1, ABCC1, and ABCG2 did not increase during the differentiation of these cells (Figure 5C). However, when HaCaT cells were stimulated with PTC or PROP, the expression of ABCB1 and ABCG2 increased (Figure 5D,E). In particular, we confirmed the enhanced production of the ABCB1 protein in these cells (Figure 5F). As shown in Figure 5G,H, the PTC or PROP‐induced expression of ABCB1 was reduced by treatment with Y‐27632 and SB203580. Moreover, we found the similar effects of SB203580 on the production of ABCB1 by the stimulation with other bitter substances, such as denatonium or saccharin (Figure S5A). Thus, the TAS2Rs/G_α12/13_/RhoA/ROCK/p38 MAPK/NF‐κB axis could be implicated in these augmentations (Figure 5G,H). Moreover, enhanced expression of ABCB1 and ABCG2 upon stimulation with PTC or PROP was observed in differentiated NHK cells (Figure 5I). A similar enhanced production of the ABCB1 protein was confirmed in these cells (Figure S5B). In addition, the production of ABCB1 was enhanced following stimulation with other bitter compounds, such as denatonium, saccharin, and salicin (Figure S5C), suggesting that bitter compounds induced the production of ABC transporters, especially ABCB1, via the TAS2Rs/G_α12/13_/RhoA/ROCK/p38 MAPK/NF‐κB axis (Figure 5J).

### 3.6 Implication of ABCB1 in the excretion of substances added to bitter compound‐exposed HaCaT cells

**FIGURE 6:**
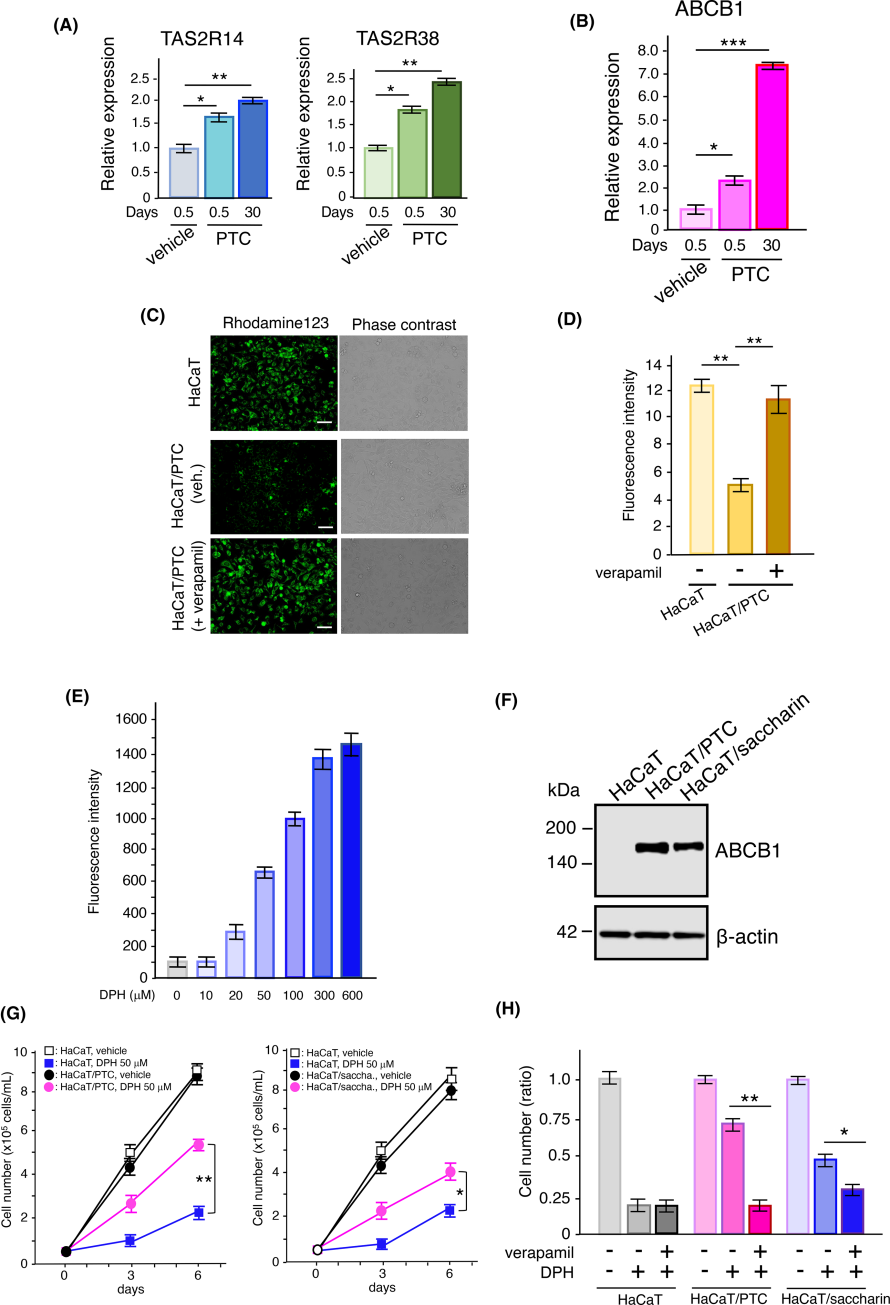
Enhanced activation of ABCB1 in bitter compound‐exposed HaCaT cells. (A) Increased expression of TAS2R14 and TAS2R38 in HaCaT cells cultured in the presence of 1 mM PTC for 0.5 or 30 days *n* = 4. (B) Increased ABCB1 expression in HaCaT cells cultivated in the presence of 1 mM PTC for 0.5 or 30 days *n* = 4. (C) Accumulation of rhodamine 123 in HaCaT and HaCaT/PTC cells. The cells (2 × 10^5^ cells) were seeded into collagen‐coated glass‐bottomed 35 mm dishes. Intracellular retention was evaluated by incubating 10 μM rhodamine 123 for 1 h in the dark at 37°C in the presence or absence of 20 μM verapamil. After the cells were washed, images were acquired using fluorescence microscopy at 488 nm excitation and 535 nm emission wavelength. Scale bar, 100 μm. (D) Accumulation of rhodamine 123 in HaCaT/PTC cells by treatment with verapamil. Fluorescence intensities were determined using the ImageJ software *n* = 5. (E) Toxicity of DPH in HaCaT cells was determined using a Zombie Red Fixable Viability Kit. After the cells were washed, their fluorescence intensities were determined using flow cytometry *n* = 4. (F) Western blot of ABCB1 in the membrane fractions from HaCaT, HaCaT/PTC, and HaCaT/saccharin cells. β‐actin was employed as an experimental and loading control. (G) Effects of DPH on cell growth. HaCaT, HaCaT/PTC, and HaCaT/saccharin cells (2 × 10^4^ cells/mL) were cultured in the presence or absence of 50 μM DPH for 6 days *n* = 6. (H) Inhibition of HaCaT/PTC and HaCaT/saccharin cell growth by verapamil. HaCaT, HaCaT/PTC, and HaCaT/saccharin cells (2 × 10^4^ cells/mL) were cultured in the presence or absence of 50 μM DPH and 20 μM verapamil, for 6 days *n* = 6. Data are expressed as mean ± SEM. *p < 0.05, **p < 0.01, ***p < 0.001 using two‐way ANOVA followed by Tukey's post hoc test (A, B, D, G, H).

To further understand the significance of the enhanced expression of ABCB1 through TAS2R activation, HaCaT/PTC cells were cultured long‐term (>30 days) in the presence of 1 mM PTC. We found further increased expression of TAS2Rs, such as TAS2R14 and TAS2R38 (Figure 6A), and ABCB1 (Figure 6B) in these cells compared to that in parental cells. Rhodamine 123 is a membrane‐permeable dye readily incorporated in living cells and rapidly excreted via ABCB1.^38^, ^39^ The accumulation of rhodamine 123 in HaCaT/PTC cells was lower than that in parental cells (Figure 6C). The fluorescence of rhodamine 123 in these cells was increased by treatment with verapamil, an inhibitor for ABCB1, suggesting the enhanced activation of ABCB1 in HaCaT/PTC cells (Figure 6D). These results suggest that long‐term exposure to ligands for TAS2Rs leads to increased ABCB1 production, resulting in enhanced excretion of substances incorporated in cells. To obtain further evidence regarding the significance of the enhanced production of ABCB1 by TAS2R activation, we examined the effect of a harmful substance on the growth of bitter compound‐exposed HaCaT cells. DPH is a bitter compound and a histamine H1 receptor antagonist that induces severe cell toxicity upon overdose.^40^, ^41^ DPH was found to exhibit dose‐dependent toxicity in parental HaCaT cells, with an IC_50_ of approximately 50 μM (Figure 6E), which is consistent with the prior results. In this experiment, the HaCaT cell line was cultured in the presence of 1 mM saccharin for over 30 days (HaCaT/saccharin cells). Prior to the analyses, the production of the ABCB1 proteins in HaCaT/PTC and HaCaT/saccharin cells was confirmed to be enhanced compared to that in parental cells (Figure 6F). In the presence of 50 μM DPH, HaCaT/PTC cells and HaCaT/saccharin cells exhibited a higher proliferation rate than parental HaCaT cells (Figure 6G). The enhanced proliferation of HaCaT/PTC and HaCaT/saccharin cells was reduced by treatment with verapamil (Figure 6H), suggesting involvement of ABCB1 in the predominant growth of these cells. Taken together, these results suggest that the TAS2Rs/G_α12/13_/RhoA/ROCK/p38 MAPK/NF‐κB/ABCB1 pathway plays a critical role in the excretion of harmful substances from cells. Moreover, activation of TAS2Rs by bitter compounds, such as PTC and saccharin, enhances ABCB1 production, leading to the prevention of DPH‐induced cell death via excretion of these compounds.

## 4 DISCUSSION

TAS2Rs recognize various chemical substances, thereby protecting humans from ingesting toxins.^3^, ^5^ According to many studies, TAS2Rs are expressed not only in taste cells but also in various tissues, including the gastrointestinal tract, lungs, kidneys, skin, and brain; however, a physiological picture of their function has not been obtained.^4^, ^9^, ^10^, ^26^, ^42^ Recent studies revealed that TAS2Rs, such as TAS2R1, TAS2R14, and TAS2R38, are expressed in human keratinocytes and HaCaT cells; For example, Shaw et al. clarified the expression of many TAS2Rs in human skin by RNA‐seq analysis, although expression levels differed markedly among individuals.^4^ However, their biological roles have not been clarified.^11^, ^12^, ^43^ Furthermore, stimulation of TAS2Rs by bitter compounds induced intracellular Ca^2+^ influx and upregulated genes involved in skin barrier function in HaCaT cells.^11^ In the present study, we confirmed the expression of 25 human TAS2Rs in keratinocytes and HaCaT cells. Notably, the expression levels of TAS2R14 and TAS2R38 in HaCaT cells were enhanced during keratinocyte differentiation and further increased by stimulation with bitter compounds. These data highlight the biological significance of TAS2Rs in keratinocytes.

The TAS2Rs were found to localize intracellularly, possibly in the ER. Recently, Kim et al. reported that TAS2R14 is predominantly trapped intracellularly in HEK293T cells and human airway smooth muscle cells.^44^ However, several studies have demonstrated that TAS2Rs mediate intracellular Ca^2+^ increases via surface receptors.^5^ The discrepancy in location between previous studies and our study could be due to differences in the TAS2R expression systems. For example, Meyerhof et al. used the pcDNA5/FRT vector system to express TAS2Rs in HEK293T cells; thus, receptor proteins were abundantly produced, resulting in the transient transport of the receptors to the cell surface, which might be due to excess accumulation in the ER.^5^ In contrast, we expressed TAS2Rs in HEK293T and HeLa cells using a moderate enhancer/promoter vector system. Thus, we investigated the lower surface locations, despite the generation of a significant amount of TAS2R protein in these cells. In this study, various cell responses were found to be elicited by treatment with bitter compounds, such as p38 MAPK and NF‐κB activation, and ABCB1 production in these cells. To validate these responses via endogenous TAS2Rs in HaCaT cells, knockdown experiments must be performed in future studies to determine the TAS2Rs responsible for these results. Several GPCRs have been found to induce cellular signaling from intracellular compartments, such as the Golgi apparatus or endosomes.^45^, ^46^, ^47^, ^48^ However, many of these receptors elicit primary signaling from the cell surface and are then translocated to intracellular compartments. TAS2Rs transduce G_α12/13_‐mediated signals from intracellular compartments, probably the ER, without being transported to the plasma membrane. These results highlight the potential of intracellular TAS2Rs to recognize various substances incorporated in cells, ultimately leading to the development of a system for excreting these compounds.

In taste cells, TAS2Rs transduce signals through G_αgust_.^25^ However, we could not detect the expression of G_αgust_ in keratinocytes and HaCaT cells. Nonetheless, G_α12/13_‐mediated cellular signals via TAS2R14 and TAS2R38 were observed in these cells. TAS2R38 is mainly responsible for human polymorphisms associated with PTC and PROP.^49^ For individuals with the TAS2R38 sensitive form (PAV‐type), the concentrations of PTC and PROP, which are neutral for the non‐taste variant (AVI‐type), are extremely bitter.^49^ In fact, we detected enhanced reporter activity upon stimulation with PTC via PAV‐type TAS2R38 but not the AVI‐type in HEK293T cells. HaCaT cells were confirmed to harbor the former type of TAS2R38 based on PCR analysis of the *TAS2R38* gene. Thus, the response detected in HaCaT cells stimulated with PTC in the reporter assay could be evoked through this receptor.

In this study, we clarified the G_α12/13_/RhoA/ROCK/p38 MAPK/NF‐κB axis as the signaling pathway via intracellular TAS2Rs in HaCaT cells. The activation of p38 MAPK via the G_α12/13_/RhoA/ROCK axis was observed in various cells.^50^, ^51^, ^52^, ^53^ For instance, activation of the G_α12/13_‐coupled angiotensin II AT_1_‐receptor induced vascular endothelial dysfunction via the RhoA/ROCK/p38 MAPK pathway.^54^ Proinflammatory cytokines and chemokines, which play crucial roles in the recruitment and activation of neutrophils and macrophages, are produced via the NF‐κB pathway in keratinocytes.^55^ For example, activation of Toll‐like receptors (TLRs), including TLR‐1, ‐2, ‐3, ‐4, ‐5, ‐6, and ‐9, is critical for the enhanced production of various cytokines and chemokines via NF‐κB in keratinocytes, leading to the initiation and amplification of inflammation after skin injury.^31^, ^56^ However, an increased expression of cytokines and chemokines was not observed in HaCaT cells stimulated with bitter compounds, such as PTC. Consistent with our results, several studies have demonstrated the involvement of the bitter compounds/TAS2Rs axis in anti‐inflammatory effects, including reduced production of proinflammatory cytokines and attenuation of immune cell chemotaxis.^57^, ^58^, ^59^, ^60^, ^61^, ^62^ Curcumin attenuates several cytokines, such as IL‐1β, IL‐6, and TNFα, in TNFα‐stimulated HaCaT cells^30^ and amarogentin exhibits anti‐inflammatory effects by reducing IL‐6, IL‐4, and IL‐13 secretion.^63^ These findings suggest that the bitter compounds/TAS2Rs axis in keratinocytes induces anti‐inflammatory effects, including the reduced production of cytokines and chemokines, despite the activation of NF‐κB, which induces the expression of ABCB1.

ABCB1 is a well‐known transporter protein involved in the export of various substances across the cell membrane.^64^, ^65^ This transporter is mainly expressed in tissues with barrier functions, such as the intestine, liver, and skin.^66^, ^67^, ^68^, ^69^ According to previous studies, NF‐κB is involved in the transcription of the *ABCB1* gene.^33^, ^34^, ^35^, ^70^ For example, NF‐κB signaling inhibitors downregulated ABCB1 expression in breast cancer cells.^36^ As keratinocytes and HaCaT cells express ABCB1,^67^, ^69^ the production of ABCB1 is expected to be augmented via the activation of NF‐κB in these cells. p38 MAPK is well known as a key player for the activation of NF‐κB.^27^ A previous study revealed that resveratrol, which is a polyphenol in various plants, downregulates the expression of ABCB1 by suppressing the p38 MAPK/NF‐κB signaling pathway in U2OS/Adriamycin cells.^28^ Indeed, we observed the inhibitory effects of the p38 MAPK blocker on NF‐κB activation and ABCB1 expression. Such finding suggests the enhanced expression of ABCB1 via the p38 MAPK/NF‐κB pathway in keratinocytes and HaCaT cells stimulated with bitter compounds.

Various substances that infiltrate cells are excreted by several ABC transporters, such as ABCB1, ABCC1, and ABCG2.^37^ As the bitter compounds activate cognate TAS2Rs, leading to enhanced production of ABCB1 in HaCaT cells, we speculate that prolonged exposure of these cells to PTC or saccharin helps defend against harmful compounds through excretion via ABCB1. Indeed, we found increased expression of ABCB1 in HaCaT/PTC and HaCaT/saccharin cells compared to parental cells. These cells exhibited rapid export of rhodamine 123 and predominant growth in the presence of a toxic dose of DPH. The predominance of HaCaT/PTC and HaCaT/saccharin cells disappeared following treatment with verapamil, proposing the enhanced excretion of harmful substances via ABCB1.

**FIGURE 7:**
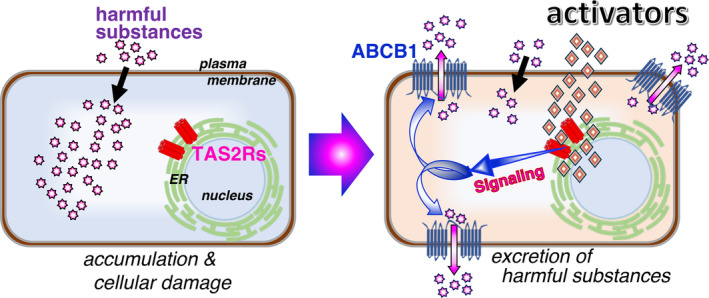
Schematic model of the excretion of harmful substances by the activation of the intracellular TAS2Rs. Activation of intracellular TAS2Rs enhances ABCB1 expression, leading to the prevention of cellular damage. For example, harmless activators of TAS2Rs may be used as protective agents against harmful substances to protect the human skin.

In summary, by the stimulation of keratinocytes with bitter compounds, the TAS2Rs/G_α12/13_/RhoA/ROCK/p38 MAPK/NF‐κB pathway was activated, and the expression of several ABC transporters, such as ABCB1, increased. An increase in ABCB1 leads to enhanced excretion of incorporated substances, including the bitter compounds. These data suggest that harmless activators of TAS2Rs may be used as protective agents against toxic substances (Figure 7). However, many TAS2Rs possess amino acid sequence polymorphisms. Thus, a significant degree of TAS2R polymorphisms may present difficulties in the development of specific and potent receptor agonists. Although more extensive studies are required to better understand the functional significance of TAS2Rs in keratinocytes, we suggest that intracellular TAS2Rs act as gatekeepers that drive the excretion of toxic substances. Therefore, we propose that TAS2Rs are potential drug targets for specific and potent receptor agonists.

## AUTHOR CONTRIBUTIONS

S.M., N.N., A.F., M.S., H.S., and M.N. performed the experiments. A.I., and J.A. constructed TGFα‐shedding assay system and established gene‐disrupted HEK293A cell lines. T.O., S.Y., and H.A. performed the experiments using keratinocytes and HaCaT cells. S.M., N.N., A.F, and M.N. performed statistical analyses. M.N. conceived and designed the study, acquired, analyzed and interpreted the data. M.N. prepared the manuscript.

## DISCLOSURES

The authors declare that they have no competing interests.

## Supporting information


Figure S1.
Figure S2.

Table S1.
Table S2.

## Data Availability

All data needed to evaluate the conclusions in the paper are presented in the manuscript or the supplemental materials. Requests for resources and reagents should be directed to and will be fulfilled by Motonao Nakamura (moto-nakamura@ous.ac.jp).
