# Supplementary material for: Intracellular TAS2Rs act as a gatekeeper for the excretion of harmful substances via ABCB1 in keratinocytes
Source: FASEB Bioadv. 2024 Aug 27;6(10):424–41. doi: 10.1096/fba.2024-00074 (PMC11452442; doi:10.1096/fba.2024-00074)
Supplement: Supplementary file 1 — Figure S1. Figure S2. [file FBA2-6-424-s001.pdf]

Supplemental materials

Intracellular TAS2Rs act as a gatekeeper for the excretion of harmful substances via ABCB1 in keratinocytes

Sazanami Mori, Natsuki Nakamura, Ayane Fuchigami, Satoshi Yoshimoto, Moe Sakakibara, Toshiyuki Ozawa, Junken Aoki, Asuka Inoue, Hayakazu Sumida, Hideya Ando and Motonao Nakamura *

* Corresponding author: Department of Bioscience, Graduate School of Life Science, Okayama University of Science, 1-1 Ridai-cho, Kita-ku, Okayama-shi, Okayama, Japan.

Tel.: +81 86 256 9541, E-mail: moto-nakamura@ous.ac.jp

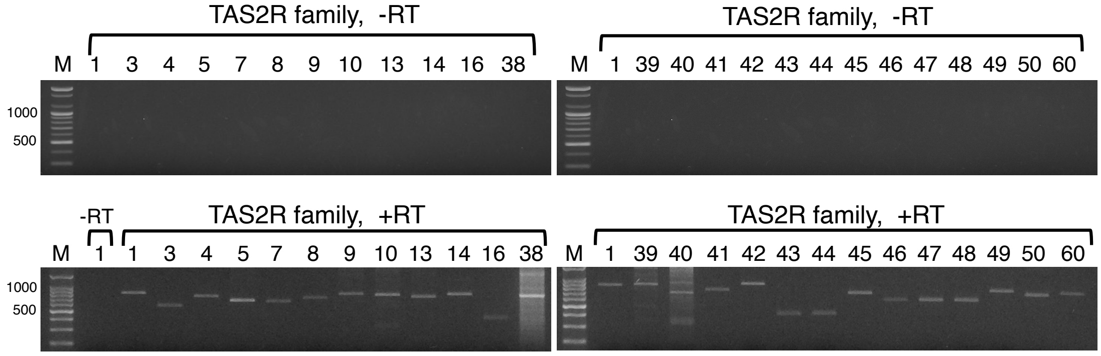

Figure S1. Expressions of TAS2Rs in HaCaT cells.

From HaCaT cells total RNAs were prepared, and RT-PCR analyses for 25 TAS2Rs were performed using these cDNAs or non-reverse transcription RNAs as templates. -RT, non-reverse transcription.

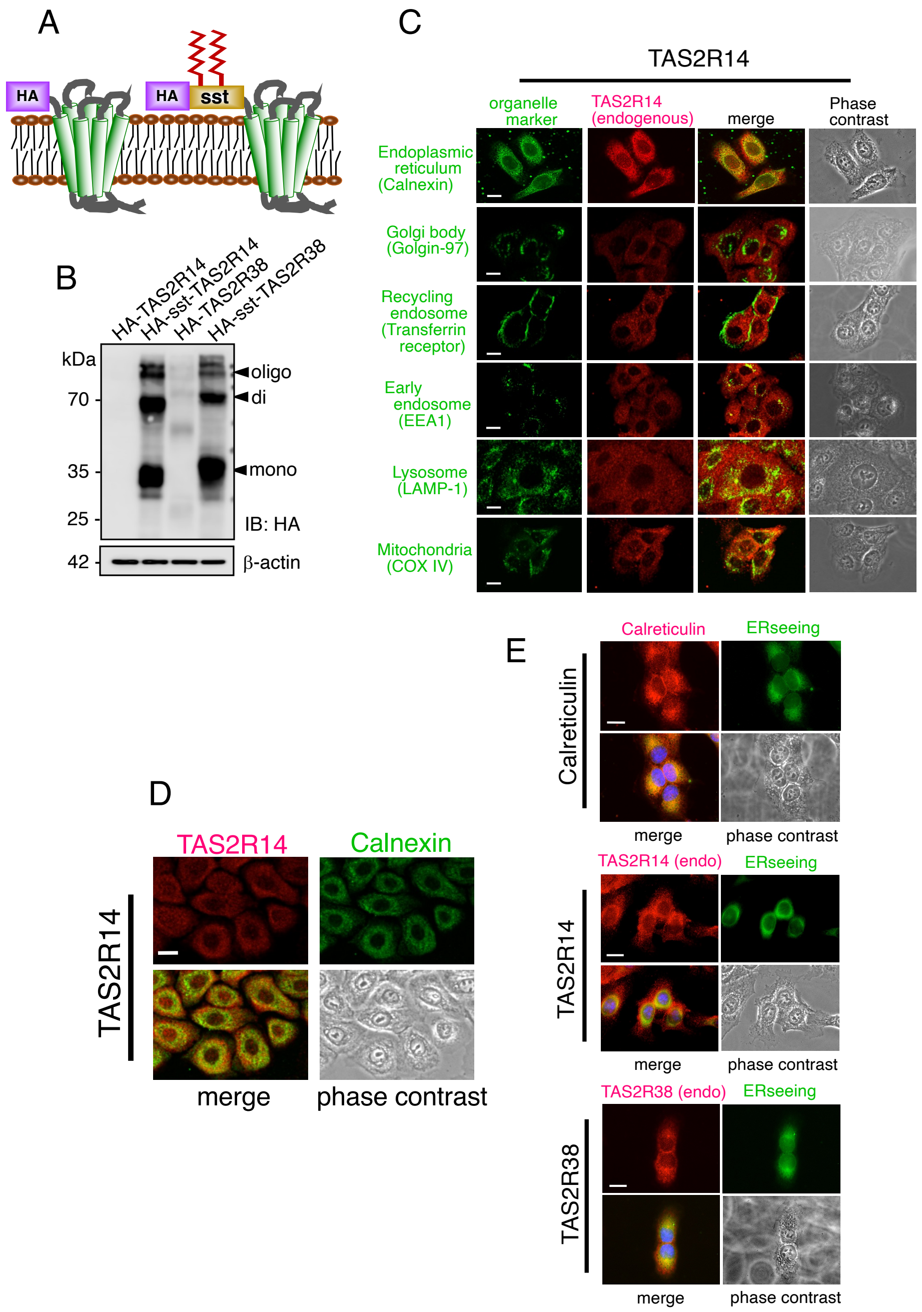

Figure S2. Intracellular localization of TAS2R14 and TAS2R38.

A, Schematic model of tagged TAS2Rs. HA, hemagglutinin; sst, 45 amino acids of rat somatostatin receptor 3. In the sst-tag region there are two N-glycosylation sites, which may be helpful for the folding of newly synthesized receptor in the ER. ***B***, Productions of receptor proteins in HEK293T cells. HEK293T cells were transfected with the indicated receptors. The membrane fractions of the cells were subjected to SDS-PAGE followed by immunoblotting for

HA-tagged or HA-sst-tagged receptors. The detected bands indicating monomer (mono), diner (di), and oligomer (oligo) of the produced receptors. β-actin is an experimental and loading control. ***C***, Immunofluorescence confocal microscopy analysis of TAS2R14 with various organelle marker proteins. HaCaT cells were subjected to immunocytochemical analysis. Organelle marker proteins (green) using anti-calnexin (ER marker), anti-golgin-97 (Golgi body marker), anti-transferrin receptor (recycling endosome marker), anti-EEA1 (early endosome marker), anti-LAMP-1(lysosome marker), and anti-COX IV (mitochondria marker) antibodies. Endogenous TAS2R14 was detected using an anti-human TAS2R14 antibody (red). Scale bar, 10 μm. ***D,*** Immunofluorescence confocal microscopy analysis of endogenous TAS2R14 in NHK cells. TAS2R14 was detected using an anti-human TAS2R14 antibody (red). Calnexin was detected using anti-calnexin antibody (green) as the ER marker. Scale bar, 10 μm. ***E***, Co-staining of endogenous TAS2R14 and TAS2R38 with ERseeing. Immunofluorescence confocal microscopy analyses of endogenous TAS2R14 and TAS2R38 with ERseeing, an ER marker. HaCaT cells were subjected to immunocytochemical analysis. Endogenous TAS2R14 (middle, red) and TAS2R38 (lower, red) were detected using an anti-human TAS2R14 and anti-TAS2R38 antibodies, respectively. Calreticulin was detected using anti-calreticulin antibody (upper, red). For the staining of the ER, 1 μM ERseeing was used. DAPI was used to stain for the nucleus (blue). endo, endogenous. Scale bar, 10 μm.

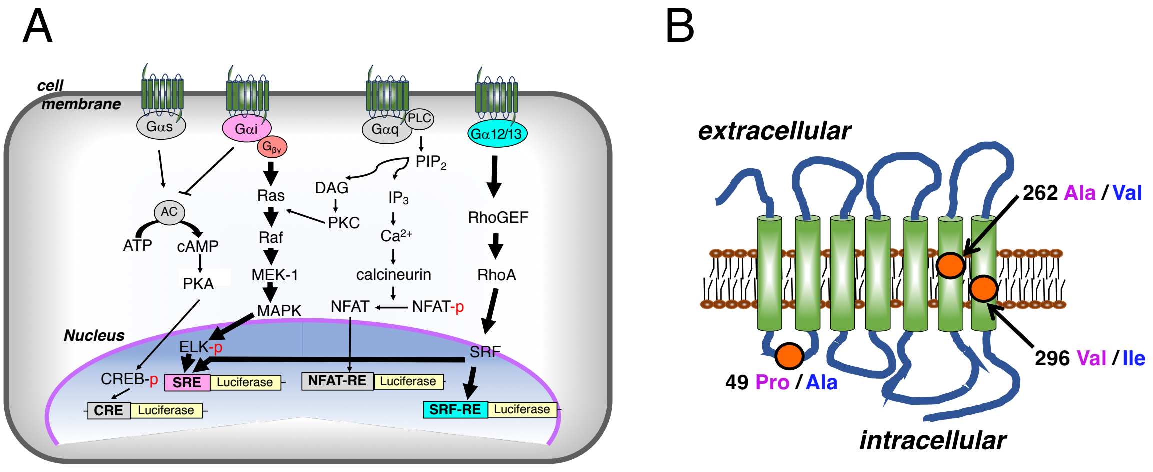

Figure S3. Coupling of intracellular TAS2R38 with $G_{\alpha 12/13}$.

A, Schematic model of the activation pathways of the reporter system. One of the 4 enhancer element, CRE, NFAT-RE, SRE, or SRF-RE, is fused to NanoLuc luciferase gene. SRE is activated via $G_{\alpha i}$ and $G_{\alpha 12/13}$, in contrast the driving of SRF-RE is regulated by only $G_{\alpha 12/13}$. ***B***, Schematic model of human TAS2R38. There are at least two types of polymorphism in TAS2R38, $Pro^{49}/Ala^{262}/Val^{296}$ (TAS2R38/PAV) and $Ala^{49}/Val^{262}/Ila^{296}$ (TAS2R38/AVI); the former is activated by PTC and PROP, whereas the latter is not.

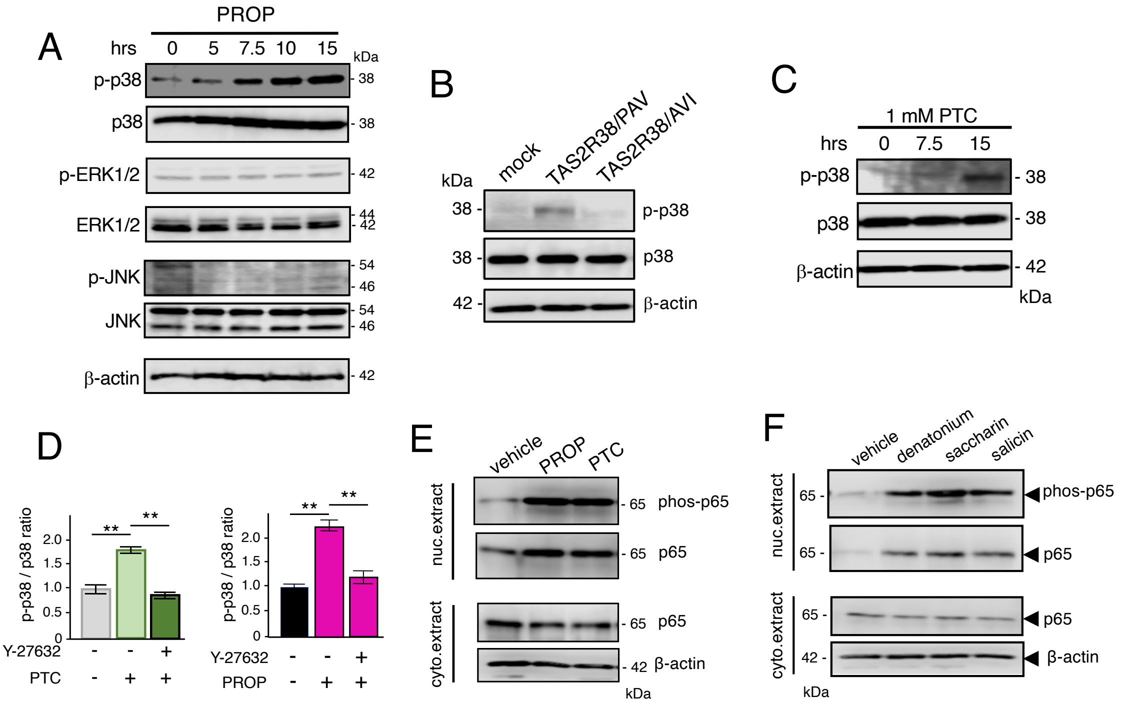

Figure S4. Activation of p38 MAP kinase/NF-κB axis.

A, Western blot showing p38 MAPK, p-p38 MAPK, ERK1/2, p-ERK1/2, JNK, and p-JNK in lysates from HEK293T cells expressing HA-sst-TAS2R38 at the indicated time points after 1 mM PROP stimulation. ***B***, Western blot showing p38 MAPK, and p-p38 MAPK in lysates from HaCaT cells expressing TAS2R38/PAV or TAS2R38/AVI after stimulation with 1 mM PTC for 15 h. ***C***, Western blot showing p38 MAPK, and p-p38 MAPK in lysates from HaCaT cells at the indicated time points after 1 mM PTC stimulation. ***D***, Reduced signal intensities of PTC (***Figure 4D***) or PROP-induced p-p38 MAPK by Y-27632 were determined by densitometric analysis. Each signal was quantified as the ration of p-p38 MAPK/p38 MAPK intensity. Data are means ± SEM. $n = 4$. $^{**}P < 0.01$ by two-way ANOVA followed by Tukey' *post hoc* test. ***E***, Western blot showing p65 and phos-p65 in cytoplasmic and nuclear extracts from HaCaT cells after stimulation with 1 mM PTC or 1 mM PROP for 15 h. ***F***, Western blot showing p65 and phos-p65 in cytoplasmic and nuclear extracts from differentiated NHK cells stimulated with 1 mM denatonium, 1 mM saccharin, or 1 mM salicin for 15 h. β-actin is an experimental and loading control.

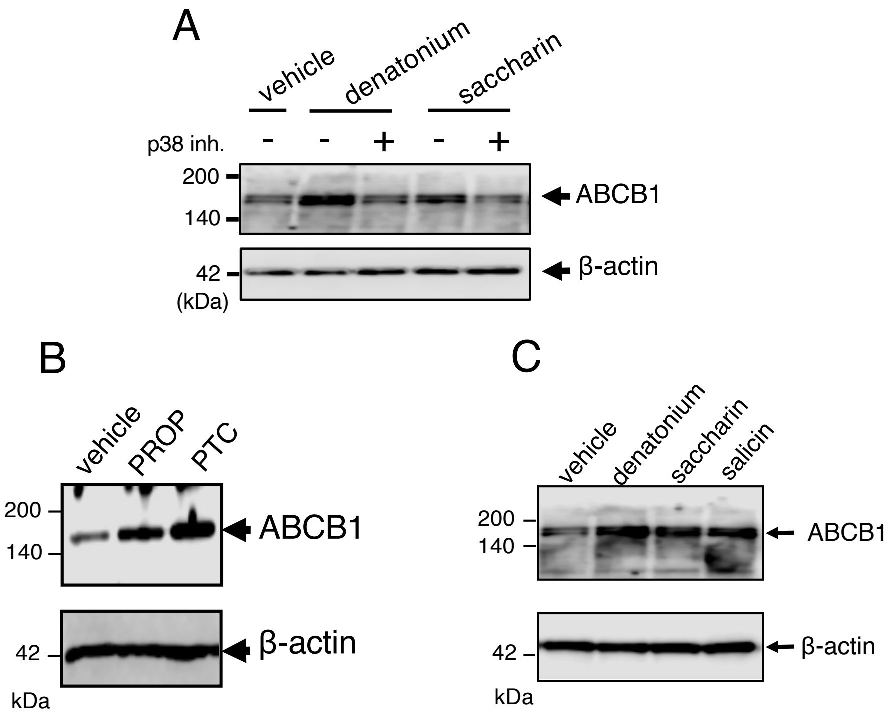

Figure S5. Enhanced expression of ABCB1 by the activation of intracellular TAS2Rs.

A, Involvement of p38 MAPK in the induction of ABCB1 production in HaCaT cells following stimulation with 1 mM denatonium or 1 mM saccharin. β-actin was employed as an experimental and loading control. ***B***, Western blot showing ABCB1 in lysates from the differentiated NHK cells stimulated with 1 mM PTC or 1 mM PROP for 15 h. ***C***, Western blot showing ABCB1 in lysates from the differentiated NHK cells stimulated with 1 mM denatonium, 1 mM saccharin, or 1 mM salicin for 15 h. β-actin is an experimental and loading control.
