# Supplementary material for: Intracellular TAS2Rs act as a gatekeeper for the excretion of harmful substances via ABCB1 in keratinocytes
Source: FASEB Bioadv. 2024 Aug 27;6(10):424–41. doi: 10.1096/fba.2024-00074 (PMC11452442; doi:10.1096/fba.2024-00074)
Supplement: Supplementary file 2 — Table S1. Table S2. [file FBA2-6-424-s002.pdf]

Supplemental materials

Intracellular TAS2Rs act as a gatekeeper for the excretion of harmful substances via ABCB1 in keratinocytes

Sazanami Mori, Natsuki Nakamura, Ayane Fuchigami, Satoshi Yoshimoto, Moe Sakakibara, Toshiyuki Ozawa, Junken Aoki, Asuka Inoue, Hayakazu Sumida, Hideya Ando and Motonao Nakamura *

* Corresponding author: Department of Bioscience, Graduate School of Life Science, Okayama University of Science, 1-1 Ridai-cho, Kita-ku, Okayama-shi, Okayama, Japan.

Tel.: +81 86 256 9541, E-mail: moto-nakamura@ous.ac.jp

Table S1. List of reagents and resources used in this study

REAGENT or RESOURSE	SOURCE	IDENTIFIER
Antibodies		
Anti-human TAS2R14	Abcam	Cat# ab138271
Anti-human TAS2R38	Abcam	Cat# ab130503
Goat Anti-Rat IgG H&L (Alexa Fluor 488)	Abcam	Cat# ab150157
Anti-b-actin Mouse mAb	SIGMA-ALDRICH	Cat# A1978
Anti-Calnexin Mouse mAb	SIGMA-ALDRICH	Cat# 047M4859V
Anti-Calreticulin Rabbit pAb	Enzo Life Sciences, Inc	Cat# ADI-SPA-600-D
Anti-CD71 (Transferrin receptor) Mouse mAb	Santa Cruz Biotechnology	Cat# sc-32272
Anti-LAMP-1（H4A3） Mouse mAb	Santa Cruz Biotechnology	Cat# sc-20011
Golgin-97 (CDF4) Mouse mAb	Cell Signaling technology	Cat# 97537
COX IV(3E11) Rabbit mAb （Alexa Fluor 488）	Cell Signaling technology	Cat# 4853s
Anti-ERK1/2 Rabbit mAb	Cell Signaling technology	Cat# 4695
Anti-phospho-ERK1/2 Rabbit mAb	Cell Signaling technology	Cat# 4370
Anti-SAPL/JNK Rabbit mAb	Cell Signaling technology	Cat# 9252
Anti-phospho-SAPK/JNK Rabbit mAb	Cell Signaling technology	Cat# 9251
Anti-p38 Rabbit mAb	Cell Signaling technology	Cat# 9212
Anti-phospho-p38 Rabbit mAb	Cell Signaling technology	Cat# 4511
Anti-MDR1/ABCB1 Rabbit mAb (D3H1Q)	Cell Signaling technology	Cat# 12683
Anti-phospho-NF-kB p65 (Ser536) Rabbit mAb	Cell Signaling technology	Cat# 3033
Anti-NF-kB p65 Rabbit mAb (D14E12)	Cell Signaling technology	Cat# 8242
Anti-EEA1 Mouse mAb	MBL	Cat# M176-3

Anti-HA High Affinity/Rat mAb (clone 3F10)	Roche Diagnostics	Cat# 11867423001
Goat Anti-Mouse IgG H&L (Alexa FluorR 488)	Invitrogen	Cat# A11001
Goat Anti-Rat IgG H&L (Alexa FluorR 546)	Invitrogen	Cat# A11081
Goat Anti-Mouse IgG H&L (Alexa FluorR 546)	Invitrogen	Cat# A11030
Goat Anti-Rabbit IgG H&L (Alexa FluorR 546)	Invitrogen	Cat# A11035
Goat F(ab')2 Anti-Rat IgG (H+L), Mouse anti body-PE	SouthernBiotech	Cat# 3052/9/1
Chemicals		
phenylthiocarbamide(PTC)	TCI	Cat# P0237
6-propyl-2-thiouracil (PROP)	TCI	Cat# P0533
denatonium benzoate	TCI	Cat# D2124
saccharine	nacalai tesque	Cat# 30432-62
salicin	nacalai tesque	Cat# 30415-34
Disodium p-Nitrophenyl phosphate Hexahydrate	Wako	Cat# 149-02342
verapamil hydrochloride	FujiFilm-Wako	Cat# 222-00781
Y-27632	FujiFilm-Wako	Cat# 030-24021
SB203580	Funakoshi	Cat# AG-CR1-0030-M001
pertussis toxin (PTX)	FujiFilm-Wako	Cat# 168-22471
ERseeing	Funakoshi	Cat# FDV-0038
VECTASHIELD Mounting Medium with DAPI	Vector Laboratories	Cat# H-1200
IntraPrep Permeabilization Reagent	BECKMAN COULTERR	Cat# A07802
Rhodamine 123	Cayman chemical	Cat# 16672
Lipofectamine™ 2000 Transfection Reagent	invitrogen	Cat# 11668019
Fura2-AM	Dojindo	Cat# 341-08621

Protease Inhibitor Cocktail for General Use	nacalai tesque	Cat# 04080-11
Kits		
Chemi-Lumi One Ultra	nacalai tesque	Cat# 11644-40
Nano-Glo^{R} Dual-Luciferase^{R} Reporter Assay System	Promega	Cat# N1610
SuperScript^{R} III First-Strand	invitrogen	Cat# 18080-051
THUNDERBIRD^{R} SYBR^{R} qPCR Mix	TOYOBO	Cat# QPS-201
RNeasy^{R}Mini Kit (250)	QIAGEN	Cat# 74106
Cell lines		
HaCaT	CLS Cell Line Servise GmbH	Cat# 300493-ACADEMIC
Norman human keratinocyte	Cascade Biologics	Cat# C00115C
HEK293T	RIKEN BioResorce Research Center	Cat# RCB2202
HEK293A	A.Inoue et al.	N/A
Gα12/13-deficient HEK293A	A.Inoue et al.	N/A
Gαi-deficient HEK293A	A.Inoue et al.	N/A
Plasmids		
pcDNA3-HA-sst-TAS2R1	This paper	N/A
pcDNA3-HA-sst-TAS2R3	This paper	N/A
pcDNA3-HA-sst-TAS2R4	This paper	N/A
pcDNA3-HA-sst-TAS2R5	This paper	N/A
pcDNA3-HA-sst-TAS2R7	This paper	N/A
pcDNA3-HA-sst-TAS2R8	This paper	N/A
pcDNA3-HA-sst-TAS2R9	This paper	N/A
pcDNA3-HA-sst-TAS2R10	This paper	N/A
pcDNA3-HA-sst-TAS2R13	This paper	N/A
pcDNA3-HA-sst-TAS2R14	This paper	N/A
pcDNA3-HA-sst-TAS2R16	This paper	N/A
pcDNA3-HA-sst-TAS2R38	This paper	N/A
pcDNA3-HA-sst-TAS2R39	This paper	N/A

pcDNA3-HA-sst-TAS2R40	This paper	N/A
pcDNA3-HA-sst-TAS2R41	This paper	N/A
pcDNA3-HA-sst-TAS2R42	This paper	N/A
pcDNA3-HA-sst-TAS2R43	This paper	N/A
pcDNA3-HA-sst-TAS2R44	This paper	N/A
pcDNA3-HA-sst-TAS2R45	This paper	N/A
pcDNA3-HA-sst-TAS2R46	This paper	N/A
pcDNA3-HA-sst-TAS2R47	This paper	N/A
pcDNA3-HA-sst-TAS2R48	This paper	N/A
pcDNA3-HA-sst-TAS2R49	This paper	N/A
pcDNA3-HA-sst-TAS2R50	This paper	N/A
pcDNA3-HA-sst-TAS2R60	This paper	N/A
pGL4.53[luc2/PGK]Vector	Promega	Cat# E501
pNL[NlucP/CRE/Hygro]Vector	Promega	Cat# CS186804
pNL[NlucP/NFAT-RE/Hygro]Vector	Promega	Cat# CS177602
pNL[NlucP/SRE/Hygro]Vector	Promega	Cat# CS177601
pNL[NlucP/SRF/Hygro]Vector	Promega	Cat# CS194101
pCAGGS-Ga12	A.Inoue et al.	N/A
pCAGGS-Ga13	A.Inoue et al.	N/A
pCAGGS-Gaq	A.Inoue et al.	N/A
pCAGGS-Gaq/s	A.Inoue et al.	N/A
pCAGGS-Gaq/i1	A.Inoue et al.	N/A
pCAGGS-Gaq/12	A.Inoue et al.	N/A
pCAGGS-Gaq/13	A.Inoue et al.	N/A
pCAGGS-AP-TGFa	S.Higashiyama et al.	N/A
pcDNA3-HA-BLT1	This paper	N/A

Table S2. List of primers for PCR

TAS2R1 (for): 5’-TGTGGTGGTGAATGGCATTG-3’
TAS2R1 (rev): 5’-CAGCACTTAACTGTGGAGGAGGAAC-3’
TAS2R3 (for): 5’-ACACATGATTCAGGGATAATGCAAA-3’
TAS2R3 (rev): 5’-TTAGCCATCTTGGTTTTTGGTAGGAAATT-3’
TAS2R4 (for): 5’-TACAGTGGTCAATTGCAAAACTTGG-3’
TAS2R4 (rev): 5’-AATGTCCTGGAGAGTAAAGGGTGG-3’
TAS2R5 (for): 5’-TGGTCCTCATATAACCTCATTATCCTGG-3’
TAS2R5 (rev): 5’-CTGCCATGAGTGTCTCTCCCA-3’
TAS2R7 (for): 5’-TGTTTTATATTGGTGCTATATCCAGATGTCTATGC-3’
TAS2R7 (rev): 5’-GGATAAATGAATGACTTGAGGGGTAGATTAGAG-3’
TAS2R8 (for): 5’-CAATTTAGTTATCGCCAGAATTTGTTTGATC-3’
TAS2R8 (rev): 5’-TTATTTAAAACAATTAAAATAAGTGAGTGACCCAAGG-3’
TAS2R9 (for): 5’-TGAATTGACCATAGGGATTTGGG-3’
TAS2R9 (rev): 5’-ATAATTAGAATGAATGAATGGCTTGATGG-3’
TAS2R10 (for): 5’-GACTTGTAAACTGCATTGACTGTGCC-3’
TAS2R10 (rev): 5’-AAAGAGGCTTGCTTTAGCTTGCTG-3’
TAS2R13 (for): 5’-GGGTCAGTAAAAGAGAGCTGTCCTC-3’
TAS2R13 (rev): 5’-ATCAGAAGAAAGGAGTGGCTTGAAG-3’
TAS2R14 (for): 5’-GCTTTGGCAATCTCTCGAATTAGC-3’
TAS2R14 (rev): 5’-CTCTAAATTCTTTGTGACCTGAGGGC-3’
TAS2R16 (for): 5’-CCTGGGAATTTTTTAATATCCTTACATTCTGGT-3’
TAS2R16 (rev): 5’-GAAGCGCGCTTTCATGCTT-3’
TAS2R38 (for): 5’-ACAGTGATTGTGTGCTGCTG-3’
TAS2R38 (rev): 5’-GCTCTCCTCAACTTGGCATT-3’
TAS2R39 (for): 5’-TGTCGCCATTTCTCATCACCTTA-3’
TAS2R39 (rev): 5’-ATTGAGTGGCTGGCAGGGTAG-3’
TAS2R40 (for): 5’-AGAGTGCATCACTGGCATCCTT-3’
TAS2R40 (rev): 5’-GAGGATGAGAAAGTAGCTGGTGGC-3’
TAS2R41 (for): 5’-GGTTGCTGCCCTTGGATATGA-3’
TAS2R41 (rev): 5’-TGAAGATGAGGATGAAGGGATGG-3’
TAS2R42 (for): 5’-ATGGCCACCGAATTGGACA-3’
TAS2R42 (rev): 5’-GCTTGCTGTTTCCCAGAATGAG-3’
TAS2R43 (for): 5’-GAGTGGTTCAAGAGACAAAAGATCTCC-3’
TAS2R43 (rev): 5’TACATTGCACTCTTCAATTTGATCTTCC-3’

TAS2R44 (for): 5’-GCTCTGGCGGTCTCCAGAGTTGG-3’
TAS2R44 (rev): 5’-GTCAGAGTGAAGGGCACTAAGTTTCC-3’
TAS2R45 (for): 5’-CTCCTTTGCTGACCAAATTGTC-3’
TAS2R45 (rev): 5’-GAACGGGTGGGCTGAAGAAC-3’
TAS2R46 (for): 5’-GAGTTGAATCCAGCTTTTAAC-3’
TAS2R46 (rev): 5’-ATAGCTGAATGCAATAGCTTC-3’
TAS2R47 (for): 5’-GGTGTTATTACTTACATTGGTATGCAACTC-3’
TAS2R47 (rev): 5’-AAGACAGGTTGCTTTTCCAGC-3’
TAS2R48 (for): 5’-GGTTTACTCTGGGTCATGTTATTC-3’
TAS2R48 (rev): 5’-TTTGCTCTGCTGTGTCCTAAG-3’
TAS2R49 (for): 5’-GCACTGATAAATTTCATTGCCTGG-3’
TAS2R49 (rev): 5’-TTGTTCCCCCAAATCAGAATGAAT-3’
TAS2R50 (for): 5’-GGTAAATTTCATTGACTGGGTGAAGAG-3’
TAS2R50 (rev): 5’-CCTTGCTAACCATGACAACTGGG-3’
TAS2R60 (for): 5’-CAGGCAATGGCTTCATCACTG-3’
TAS2R60 (rev): 5’-TCCCACACCCAGAATTTAAAGTC-3’

Table S3. List of primers for quantitative PCR

q-IL1β (for): 5’-CCACAGACCTTCCAGGAGAATG-3’
q-IL1β (rev): 5’-GTGCAGTTCAGTGATCGTACAGG-3’
q-IL6 (for): 5’-AGACAGCCACTCACCTCTTCAG-3’
q-IL6 (rev): 5’-TTCTGCCAGTGCCTCTTTGCTG-3’
q-IL8 (for): 5’-GAGAGTGATTGAGAGTGGACCAC-3’
q-IL8 (rev): 5’-CACAACCCTCTGCACCCAGTTT-3’
q-TNFα (for): 5’-CTCTTCTGCCTGCTGCACTTTG-3’
q-TNFα (rev): 5’-ATGGGCTACAGGCTTGTCACTC-3’
q-CXCL1(for): 5’-AGCTTGCCTCAATCCTGCATCC-3’
q-CXCL1 (rev): 5’-TCCTTCAGGAACAGCCACCAGT-3’
q-CXCL9 (for): 5’-CTGTTCCTGCATCAGCACCAAC-3’
q-CXCL9 (rev): 5’-TGAACTCCATTCTTCAGTGTAGCA-3’
q-TAS2R14 (for): 5’-CATGTGGAAACATCGCAAGAA-3’
q-TAS2R14 (rev): 5’-GCTGGCGTCTCCGGATATT-3’
q-TAS2R38 (for): 5’-AGCTGGGTCTCCAGGAAGATC-3’
q-TAS2R38 (rev): 5’-GCAGATGCAGGAGCAAAGAAT-3’
q-ABCB1 (for): 5’-CTCATCGTTTGTCTACAGTTCGT-3’
q-ABCB1 (rev): 5’-ACAATGACTCCATCATCGAAACC-3’
q-ABCC1 (for): 5’-TGCTGCACCAGTACTTCCACAT-3’
q-ABCC1 (rev): 5’-CCCCAATGACAGCGGTCTT-3’
q-ABCG2 (for): 5’-GCAGCTCTTCGGCTTGCA-3’
q-ABCG2 (rev): 5’-CCCTGTTAATCCGTTCGTTTTT-3’
q-actin (for): 5’-CAGGATGCAGAAGGAGATCACTG-3’
q-actin (rev): 5’-TACTCCTGCTTGCTGATCCACAT-3’

Table S4. Native TAS2R38 genotypes

genotype		AVI	PAV	PAI	PVI
cell line		49-g(Ala)<br>262-t(Val)<br>296-a(Ile)	49-c(Pro)<br>262-c(Ala)<br>296-g(Val)	49-c(Pro)<br>262-c(Ala)<br>296-a(Ile)	49-c(Pro)<br>262-t(Val)<br>296-a(Ile)
HL60		○			○
HaCaT			○		○
Caco2		○	○		
HeLa		○			
HEK293		○	○		
HSC-2			○	○	
HSC-3			○		
OSC-19		○			
Susa		○			
NHK		○	○		
